# Chemical Gradients in Polymer-Modified Paper Sheets—Towards Single-Layer Biomimetic Soft Robots

**DOI:** 10.3390/biomimetics8010043

**Published:** 2023-01-18

**Authors:** Jan-Lukas Schäfer, Tobias Meckel, Simon Poppinga, Markus Biesalski

**Affiliations:** 1 Department of Chemistry, Macromolecular Chemistry & Paper Chemistry, Technical University of Darmstadt, Alarich-Weiss-Straße 8, 64287 Darmstadt, Germany; 2 Department of Biology, Botanical Garden, Technical University of Darmstadt, Schnittspahnstraße 10, 64287 Darmstadt, Germany

**Keywords:** cellulose, fiber, sheet, polymer adsorption, humidity actuated devices, directed transport, wet strength, biomimetics

## Abstract

Biomimetic actuators are typically constructed as functional bi- or multilayers, where actuating and resistance layers together dictate bending responses upon triggering by environmental stimuli. Inspired by motile plant structures, like the stems of the false rose of Jericho (*Selaginella lepidophylla*), we introduce polymer-modified paper sheets that can act as soft robotic single-layer actuators capable of hygro-responsive bending reactions. A tailored gradient modification of the paper sheet along its thickness entails increased dry and wet tensile strength and allows at the same time for hygro-responsiveness. For the fabrication of such single-layer paper devices, the adsorption behavior of a cross-linkable polymer to cellulose fiber networks was first evaluated. By using different concentrations and drying procedures fine-tuned polymer gradients throughout the thickness can be achieved. Due to the covalent cross-linking of polymer with fibers, these paper samples possess significantly increased dry and wet tensile strength properties. We furthermore investigated these gradient papers with respect to a mechanical deflection during humidity cycling. The highest humidity sensitivity is achieved using eucalyptus paper with a grammage of 150 g m^−2^ modified with the polymer dissolved in IPA (~13 wt%) possessing a polymer gradient. Our study presents a straightforward approach for the design of novel hygroscopic, paper-based single-layer actuators, which have a high potential for diverse soft robotic and sensor applications.

## 1. Introduction

During recent years, a variety of biomimetic, self-sufficient, and functionally robust actuators have been developed for applications in, e.g., soft robotics, medicine, and architecture (reviewed by [1,2]). They are based on an interplay of several material layers, which react differentially to environmental stimuli such as changes in humidity. By this, they represent functional analogies to natural actuators such as pinecones, which open when it is dry (entailing seed dispersal by wind) and close when it is wet (thereby keeping the seeds safe). This behavior of the cones is rendered possible by the differential swelling and shrinking properties of the functionally relevant tissue layers inside the individual pinecone scales [3,4]. The mentioned biomimetic compliant mechanisms, which function without the need for typical hinges as otherwise found in rigid-body-mechanisms [5], can be constructed from a multitude of different natural and/or technical materials systems (reviewed by [6]).

In this context, paper materials offer a variety of benefits as they are fully recyclable and originate from biogenic polymeric resources. Due to its nature, paper can be used as a versatile platform for a variety of applications, such as microfluidic devices like lateral flow assays for the rapid detection of different molecules in analytes [7,8], devices for sensing strain, gas, and humidity [9,10], and self-actuated devices that are driven by changes in temperature, light, or humidity [11,12]. Most of these soft robotic devices are fabricated by modifying the paper with, e.g., stimuli-responsive polymers, small molecules, or inorganic particles in order to achieve the desired properties [13]. Techniques that are commonly used for fabrication include printing, handwriting, coating, impregnation, precipitation, and grafting polymerization. However, studies where paper is used as an integral, motion-inducing, and influencing part of the soft robot and not only as a carrier and/or substrate are very rare [14,15,16].

The mechanical properties, in particular the wet strength, are important parameters for humidity-actuated devices. Combining spatially resolved multifunctional modifications in the paper that lead to self-actuation properties and at the same time increase the wet strength would be highly beneficial but have not been reported to the best of our knowledge yet.

Dry and wet strength agents are commonly applied in the wet end of industrial paper making, i.e., mixed with the fiber pulp suspension before the fibers are laid down in a non-woven fashion. Here, the amount of additive can be controlled by adjusting the concentration of the additive in the feed [17]. The development of the dry tensile strength of paper with an increasing amount of added cationic polyacrylamide (CPAM) in the wet end is a well-studied phenomenon that can be described with a saturation curve (see, e.g., [18]). The spatial distribution of CPAM in the fiber network and the fibers themselves and the resulting tensile properties have been investigated on macroscopic and microscopic scales [19,20]. It was observed that CPAM added in the wet end (internal application) or by impregnation (external application) was homogeneously distributed throughout the paper thickness. However, for the single- and double-sided coatings, CPAM is only enriched at the top surface or both at the top and the bottom surfaces, respectively, with less in the bulk. The dry tensile properties also varied with the application procedure, showing a plateau for the wet end application, as described above. Remarkably, the same plateau was not observed for impregnation or the application by coating, where the tensile index values increased throughout the whole investigated range of CPAM addition. For the widely used wet strength agent polyamidoamine epichlorohydrin (PAE), similar saturation curves and plateauing wet tensile values were found in a number of studies, e.g., [21,22,23]. Obokata and Isogai [21] investigated the internal and external addition of PAE to paper samples but were unable to observe any differences in wet tensile strengths, which, however, might be attributable to their small sample size. Attempts have been made to study the spatial distribution of PAE in paper sheets; however, these were constrained by spatial resolution due to the use of FT-IR spectroscopic imaging [24] or transmitted light microscopy [25]. Therefore, the focus was primarily on the spatial distribution on the macroscopic scale until now.

The diffusion of macromolecules inside the pores of cellulosic fibers has been studied extensively and is regarded as quite complex [18,26,27,28]. However, uncharged polymers have not been the focus of these studies. Horvath et al. [29] showed that the adsorption kinetics of native (uncharged) dextran is significantly influenced by the molecular weight. Furthermore, they conclude that, as the diffusion of polymers in a porous medium is governed by a reptation-like process, this dependence can be explained by hydrodynamic interactions of macromolecules with the pore wall. Hoffmann et al. [26] investigated the adsorption of the uncharged cellulose derivative methyl hydroxyethyl cellulose (MHEC) into samples of unmodified cotton (fibers) and found relatively little adsorption in comparison to macromolecules with multiple cationic or anionic charges. One possible explanation mentioned in their work is that the MHEC adsorption is impacted primarily by hydrogen bonding and hydrophobic interactions, while the adsorption of charged macromolecules is governed by electrostatic interactions and the release of counterions in the (charged) cellulosic material.

Recently, the fabrication of Janus-type paper with fine-tuned silica gradients across the paper thickness was reported [30]. The gradients were achieved by utilizing a thermal drying step with directed evaporation. Based on these findings it can be assumed that the adsorption of an uncharged polymer in solution in a fiber network can be controlled by adjusting the application parameters. By precisely choosing the temperature and concentration during application, the transport of the solvent and the adsorbent can be controlled and distinct gradients throughout the paper thickness achieved. Any modification of paper fibers, and in particular if fibers are being cross-linked as discussed above, can change the mechanical properties of the resulting material. The tensile properties of inhomogeneously distributed strength additives; however, have only been the focus of a few studies (see [19,20]). However, for any development of soft robotic single-layer paper devices that exhibit humidity-activated deflection, thereby mimicking systems from nature, it is of utmost importance to also investigate and understand how gradient modification of paper sheets affects the tensile properties.

In our previous study [31] we showed that the spatial distribution of an uncharged photo-cross-linkable copolymer is significantly influenced by the choice of solvent used in the modification step. This was mainly attributed to the different abilities of the solvents to swell cellulose fibers, as reported by [32]. Furthermore, the tensile properties also differed depending on the spatial distribution. This copolymer system opens the possibility to gain more insight into the mechanisms of the adsorption and transport of uncharged macromolecules inside a fiber network, which has, to the best of our knowledge, not been the focus of previous studies.

In the present contribution, we extend our previous findings by assessing the adsorption dynamics of the photo-cross-linkable copolymer. For this, eucalyptus paper samples were modified with the copolymer from solutions of H_2_O and isopropanol (IPA) with different concentrations, afterward assessing the macroscopic spatial distribution throughout the thickness of the samples. Additionally, freeze-drying was used to analyze the transport of the polymer in the fiber network during drying. The dry and wet tensile properties of the modified paper samples were analyzed, compared, and discussed. Finally, the first attempts were made to construct a paper-based single-layer actuation device that shows stimuli-responsive behavior in changing relative humidity environments. A model is proposed that explains the observations and enables the prediction of the behavior of (specifically) designed single-layer paper actuators in the future.

## 2. Materials and Methods

### 2.1. Materials

The chemicals and solvents used were purchased from Merck (Rove, NJ, USA), Alfa Aesar (Haverill, MA, USA), Alberdingk Boley (Greensboro, NC, USA), Fisher Scientific (Hampton, NH, USA), Fluka, Covestro (Leverkusen, Germany), and TIB Chemicals (Mannheim, Germany), respectively. Unless otherwise specified, they were used as received. For sample modification by impregnation and the extraction procedure pure distilled water was used, which is denoted as H_2_O in the following.

### 2.2. Copolymer Synthesis

The two photo-cross-linking copolymers poly(dimethylacrylamide-co-4-benzoylphenyl-2-methacrylate-co-rhodamineB-methacrylic acid (P(DMAA-co-MABP-co-RhBMA)) and poly(dimethylacrylamide-co-4-benzoylphenyl-2-methacrylate (P(DMAA-co-MABP)) were synthesized according to our previous work [31]. In brief, the monomers 4-benzoylphenyl methacrylate (MABP) and rhodamine B methacrylamide (RhBMa) were prepared according to literature and subsequently copolymerized with the matrix monomer N,N-dimethylacrylamide (DMAA). ^1^H-NMR (see Figure S1 for supporting information) was used to analyze the chemical structure and estimate the ratio of the monomers, which was observed to be about 97.7 mol% of the matrix DMAA, and 2.3 mol% of MABP and RhBMA, respectively.

The molar mass was analyzed by size exclusion chromatography (SEC) with a GRAM VS/GRAM linear 10 HS/100 µL (DMF 0.002 LiCl) column and a narrowly dispersed poly(methyl methacrylate) standard. This analysis showed that the copolymer had a number average molecular weight of around M_n_ = 31,000 g mol^−1^ (Đ ~ 5.7). The copolymer was stored inside a plastic container in a refrigerator at 6 °C until further use.

### 2.3. Model Paper Handsheet Preparation

Hand sheets for testing and modification were lab-engineered using bleached eucalyptus sulfate pulp (median fiber length (length-weighted): 0.76 mm; curl: 15.9%; fibrillation degree: 5.1%; fines content: 9.1%). The paper samples having a grammage of 80 ± 1.6, 150 ± 3.0 and 200 ± 4.0 g m^−2^, respectively, were prepared using a Rapid–Köthen sheet former according to DIN 54358 and ISO 5269/2 (REF: ISO 5269-2:2004(E), Pulps—Preparation of Laboratory Sheets for Physical Testing—Part 2: Rapid Köthen method, 2004.) In order to prevent any influence on the physical properties other than that of the copolymer, no additives or filler materials were used. Prior to impregnation, the paper was conditioned for at least 24 h under standard conditions (23 °C, 50% r.h.).

### 2.4. Impregnation of Paper Samples with Copolymer

The copolymer application by impregnation of paper samples was described in our previous work [31]. In brief, the paper samples (120 × 15 mm) were individually weighed before the procedure (m_before), impregnated in the copolymer solution with the desired concentration and solvent (dist. H_2_O (H_2_O) or isopropanol (IPA)) dried overnight, pressed to achieve flat samples, illuminated by UV-light (Newport 1000W Oriel Flood Exposure Source, λ = 365 nm; energy density E = 16 J cm^−2^), extracted with cold H_2_O, dried overnight and pressed again, before being acclimated under climate-controlled conditions (23 °C and 50% r.h.). After tensile testing, the torn samples were dried in an oven at 120 °C overnight and stored under climate-controlled conditions for a minimum of 7 days. Afterward, the weight of each paper sample was determined individually (m_after) and the adsorbed amount of copolymer in relation to the weight of the unmodified paper samples was determined:

(1) $$copolymer\%=\frac{m_{after}}{m_{before}},$$

In order to analyze the copolymer transport during drying, paper samples (15 × 15 mm) were impregnated in copolymer solutions of two different solvents, H_2_O and IPA with concentrations of 3.9 mg mL^−1^ and 17 mg mL^−1^, respectively. After impregnation, the samples were freeze-dried in l. N_2_ to stop the drying process after varying times. Freeze-drying of H_2_O-impregnated paper samples was carried out in a commercial freeze dryer (Christ Martin Alpha 1-2 LD (Christ, Osterode, Germany). In contrast, IPA-impregnated samples were freeze-dried in a flask under a strong vacuum (*p* < 10^−3^ mbar) cooled with l. N_2_ from the outside. After freeze-drying, the samples were treated according to the procedure described above after the impregnation and drying step.

### 2.5. Characterization of Modified Paper Sheets by Fluorescence Confocal Laser Scanning Microscopy

The preparation of paper samples for fluorescence confocal laser scanning microscopy (CLSM) was done in accordance with our previous work [31]. In brief, paper sheets were stained in a 100 µM aqueous Fluorescent Brightener 28 (FB) solution for ten minutes, consecutively washed in H_2_O and dried overnight. Embedding was carried out using a commercially available polyurethane system consisting of an aliphatic polyisocyanate (Desmodur N3200), a polyol (Albodur 956), and a catalyst (TIB Kat 318) in a ratio of 1:1:5 × 10^−4^. After a few vacuum cycles at room temperature, the embedded samples were cured overnight and consecutively cut into about 100 µm slices using a microtome. By embedding paper samples and analyzing their cross-section, axial resolution loss is avoided. In addition, the contribution of scatter to the final image quality is greatly reduced. This technique has been used to analyze the spatial modification of paper and the functionalization of fiber surfaces, where details can be found in recent publications [30,31,33,34]. Confocal recordings were taken with a Leica TCS SP8 (Leica Microsystems, Mannheim, Germany).

### 2.6. Characterization of Modified Paper Sheets by Tensile Strength Analysis

The physical properties of the paper samples in the dry and wet state were determined as an average of at least five samples according to DIN ISO 1924-2 with a *Zwick Z1.0* with a 1 kN and 20 N load cell, respectively, using the software *testXpert II V3.71* (*ZwickRoell GmbH & Co. Kg, Ulm, Germany*) in a controlled environment with 23 °C and 50% rel. humidity. For wet tensile strength analysis, the paper samples were submerged in H_2_O for at least five minutes, after which the excess water was removed by sandwiching between tissue paper, prior to testing analogously. The paper formation industry is using the relative wet strength as an important value, which can be calculated according to this equation:

(2) $$rel.\;wet\;strength=\frac{wet\;tensile\;index}{dry\;tensile\;index}\times 100,$$

The definition of the dry/wet tensile index is shown in the following equation:

(3) $$dry/wet\;tensile\;index=\frac{F_{max}}{b\times grammage}\times 100,$$

Here, *F_max_* is the maximum force at the break in N, *b* is the width of the sample in m, and the grammage of the paper is given in g m^−2^.

### 2.7. Actuation Experiments

Paper samples (120 × 15 mm) of high grammage, namely 150 (~280 µm) and 200 g m^−2^ (~350 µm), were impregnated with the copolymer dissolved in H_2_O (34 mg mL^−1^) and IPA (48 mg mL^−1^) for 1 min. Afterward, the H_2_O-impregnated samples were put on a Teflon plate and dried in an oven at 120 °C for 0.5 h. The IPA-impregnated samples were dried lying flat on a Teflon surface without additional heating. After drying the samples were treated in analogy to the procedure described above. These paper samples were subsequently analyzed regarding their actuation behavior in different relative humidity environments.

**Figure 1:**
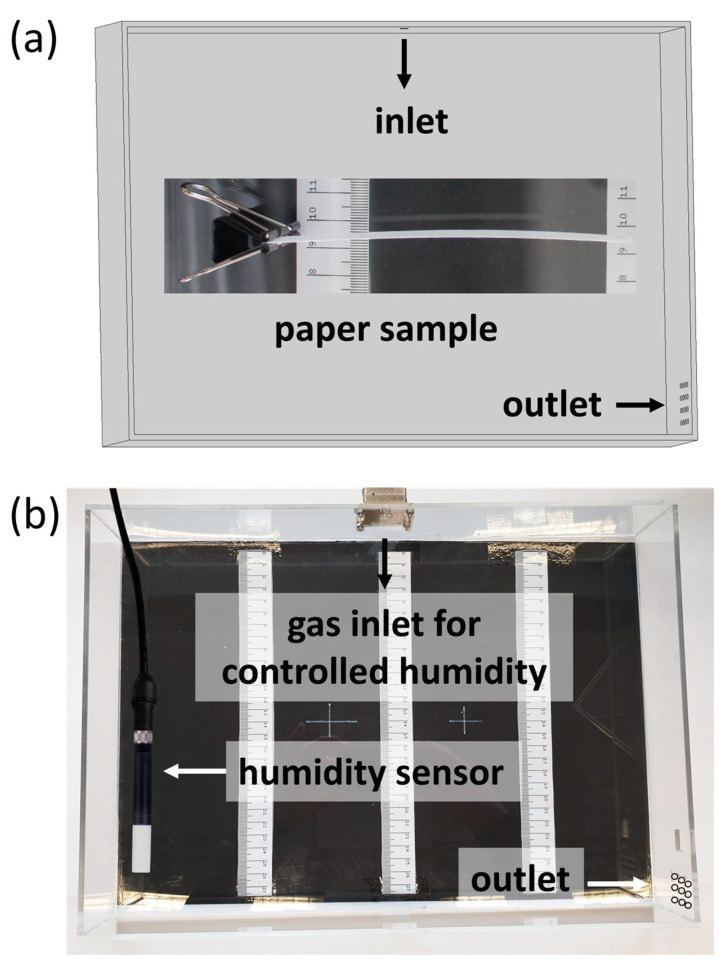
(**a**) Schematic and simplified sketch of the humidity chamber with the inlet and outlet of the air and a paper sample fixed with a clamp, standing upright in the chamber during humidity actuation experiments; (**b**) chamber built out of PMMA panels, connected to a humidity controller.

In Figure 1, the self-built climate box with humidity control (SolGelWay) is shown that was used for the humidity actuation experiments. A consumer camera (Canon EOS 600D) was used to capture images of the samples at varying humidity and rulers for the evaluation of the deflection. In order to rule out any effects of gravity on the deflection, the paper samples were fixed on one end, standing upright during the experiments.

## 3. Results and Discussion

### 3.1. Influence of Concentration and Solvent of Copolymer-Solution in Paper Samples on the Copolymer Distribution

First, we investigated the influence of different concentrations of the copolymer solutions from H_2_O and IPA on the amount of cross-linked copolymer and the resulting spatial distribution inside the paper samples. For this, a wide range of concentrations was used to impregnate paper sheets with the photo-cross-linkable copolymer out of H_2_O and IPA. For each concentration, the amount of deposited copolymer in the samples was measured gravimetrically and the spatial distribution was analyzed via CLSM.

**Figure 2:**
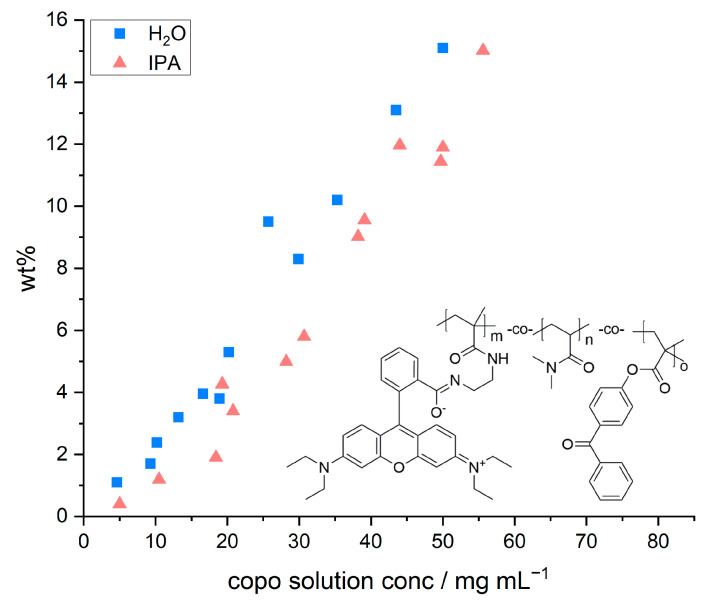
Gravimetrically determined amount of copolymer (compared to the paper dry weight) plotted against the concentration of the copolymer solution used for impregnating the paper samples out of H_2_O (blue squares) and IPA (pink triangles), respectively. The schematic structure of the used copolymer poly(dimethylacrylamide-co-4-benzoylphenyl-2-methacrylate-co-rhodamine B-methacrylic acid (P(DMAA-co-MABP-co-RhBMA)) is shown as an inset.

Figure 2 depicts the relative mass increase of the model paper sheets as a function of the concentration and the chemical structure of the copolymer, respectively. The concentration of the dip-coating polymer was chosen between 5 and 55 mg mL^−1^. Higher concentrations were not studied due to the otherwise resulting high viscosities. With both solvents used, the increase in mass followed a linear relation with increasing polymer concentration, regardless of the solvent used. The observed linear trends are in agreement with previous studies by [35], who observed a linear relationship when impregnating paper samples with PMMA solutions of different concentrations (out of tetrahydrofuran as coating solvent).

However, we also observed that the amount of adsorbed polymer was slightly higher once water was used as a solvent in comparison to IPA. These slight differences in relative weight gain may be explained by the significantly different ability of the solvents to swell cellulose fibers, alluded to earlier. Using a gravimetric approach, El Seoud et al. [32] determined quantitative values, which show that H_2_O (~62.7%) has a significantly higher swelling ability compared to IPA (~4.7%). Therefore, the overall volume taken up by the paper sheet during impregnation is slightly higher in water. Impregnating paper samples out of aqueous copolymer solutions thus leads to a significantly higher uptake of the solution and a higher amount of copolymer that can be adsorbed and cross-linked. The latter thus explains the higher values of relative mass uptake in such samples as shown in Figure 2.

**Figure 3:**
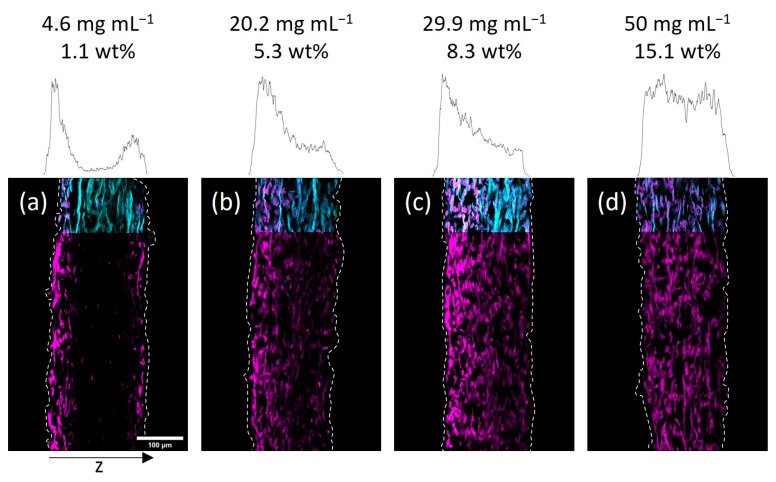
Cross-sections of paper samples modified with the photo-cross-linkable copolymer dissolved in H_2_O at different concentrations as indicated, acquired by fluorescence CLSM. The fibers are stained with Fluorescent Brightener 28 (FB, cyan color) and the copolymer fluoresces due to the rhodamine B label (magenta). The left side of the images corresponds to the side that was facing upwards during the drying and UV-excitation of samples. The z-distribution of the copolymer fluorescence is shown above each cross-section. For easier comparison, the corresponding concentrations of polymer solutions and the gravimetrically determined amount of copolymer are also noted: 4.6 mg mL^−1^, 1.1 wt% (**a**); 20.2 mg mL^−1^, 5.3 wt% (**b**); 29.9 mg mL^−1^, 8.3 wt% (**c**); 50 mg mL^−1^, 15.1 wt% (**d**).

**Figure 4:**
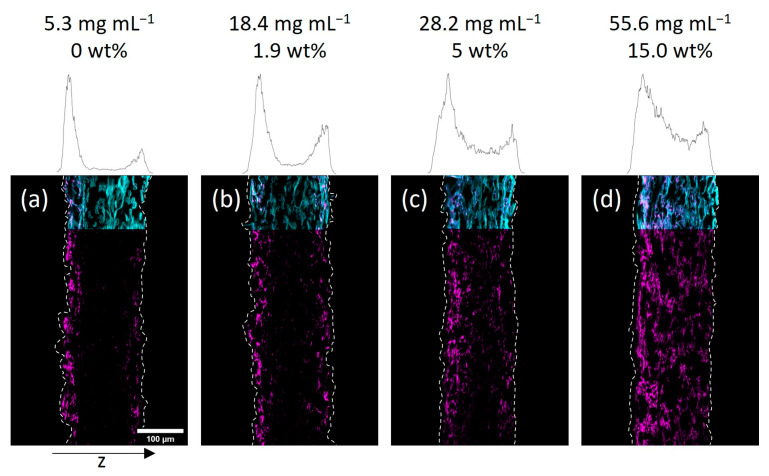
Cross-sections of paper samples modified with the photo-cross-linkable copolymer dissolved in IPA at different concentrations as indicated, acquired by fluorescence CLSM. The fibers are stained with FB (cyan color) and the copolymer fluoresces due to the rhodamine B label (magenta). The left side of the images corresponds to the side that was facing upwards during drying and UV-excitation of samples. The z-distribution of the copolymer fluorescence is shown above each cross-section. For easier comparison, the corresponding concentrations of polymer solutions and the gravimetrically determined amount of copolymer are also noted: 5.3 mg mL^−1^, 0 wt% (**a**); 18.4 mg mL^−1^, 1.9 wt% (**b**); 28.2 mg mL^−1^, 5 wt% (**c**); 55.6 mg mL^−1^, 15.0 wt% (**d**).

Next, we investigated the spatial distribution of the polymer-impregnated and cross-linked paper samples by cross-section analysis via CLSM. In Figure 3 and Figure 4 the embedded and cut paper samples modified with different amounts of the fiber-cross-linked copolymer by impregnation from H_2_O and IPA are shown, respectively. Qualitatively, it seems that a homogeneous copolymer distribution over the z-axis was achievable only at high enough concentrations of the copolymer solution. For the lowest concentrations the majority of the fluorescence signal, indicating the copolymer, was observed in the periphery of the paper samples close to their surfaces. More specifically, the side of the paper facing upwards during the drying and UV-excitation step of the modification procedure exhibits higher amounts of copolymer if compared to the opposite side. If the concentration of the polymer-concentration was increased, this effect vanishes leading to a more homogeneous distribution of the copolymer throughout the samples. Note, the latter was observed regardless of the solvent used in the impregnation step.

Taking a closer look at the CLSM data, the gradient in polymer distribution along the z-axis was significantly more pronounced for IPA-modified samples when compared to water-impregnated samples. It was observed that at the lowest concentration (see Figure 4a) the adsorption of copolymer basically only took place on the upward-facing side of the paper (left side of images). Increasing the concentration yields a sandwich-like polymer distribution with small amounts of the macromolecules being observed in the middle of the paper and the majority of the fluorescence in close vicinity to the surfaces of the sheets. The H_2_O-impregnated samples showed a similar distribution of copolymer at the lowest concentration (see Figure 3a), where transport phenomena influence the final deposition of the macromolecules. However, increasing the concentration led to a significantly more homogeneous distribution compared to the IPA-modified samples.

In order to understand this finding in more detail, we analyzed and compared the gravimetrically determined amount of copolymer in the respective samples. As we discussed earlier, the uptake and cross-linking of the polymer inside a paper sheet are slightly higher when an aqueous solution is used, compared to a solution where the polymer is dissolved in IPA. Thus, it is impossible to compare the spatial distribution of polymer inside two paper samples impregnated with solutions of equal polymer concentrations using different solvents. However, the determined amount of copolymer can be used for the comparison. For easier comparison, the CLSM images in Figure 3 and Figure 4 state the concentration of the used polymer solution, as well as the gravimetrically determined amount of polymer in the paper samples. Comparing the CLSM data of paper samples, it becomes apparent that the polymer distribution in the z-direction, indicated by the fluorescence of the copolymer (seen in magenta color), is comparable for similar polymer amounts.

Taking a closer look at the microscopic distribution of polymer in the fibers in Figures S2 and S3, a couple of interesting observations can be made. If the polymer was applied from H_2_O, the macromolecules were able to completely diffuse into the fibers, fiber walls, and lumen, respectively, leading to a homogeneous distribution of the fiber-bound polymers. If the copolymer was applied from IPA, the macromolecules seem to have only diffused into the macropores of the fiber network, leading to accumulation at the fiber–fiber-crossings. Occasionally, the copolymer was observed in the fiber lumen. Note that this qualitative finding has recently been reported in one of our previous studies [31]. The differences in the accessibility of polymer inside the fibers can be explained by a variety of factors. The lumen can be accessed through nanopores in the fiber walls, pit holes/openings in the µm-range, and either side of the fibers that could be cut open [36,37,38], which is also significantly influenced by the solvent-dependent fiber swelling [32,39]. Furthermore, the higher vapor pressure of IPA leads to faster evaporation, explaining the polymer accumulations between adjacent fibers, which are cross-linked at these sites after drying.

The importance of the transport phenomena of materials and a thermal drying step combined with directed evaporation of the impregnation solvent on the outcome of the impregnation of paper sheets has recently been reported by [30]. Paper samples were chemically modified with a silica-coating out of an ethanol–water-based solution of tetraethoxysilane (TEOS) using different drying protocols. Using a vacuum oven they observed very fast evaporation of the solvent, yielding a pronounced gradient across the z-axis with little amounts of fiber-attached silica in the center of the sheet and most of the silica adsorbing at both sides of the sheet. Depending on the concentration of the TEOS used, this gradient could even be fine-tuned. If the solvent is evaporated preferably at one side of the sheet, transport to this surface finally yields a gradient with low amounts of the fiber-attached polymer at the opposite side. Comparing our results presented here, we do observe some interesting similarities.

Apparently, the copolymer was transported within the paper sheet during the drying phase, which affected the resulting z-distribution of fiber-attached macromolecules after cross-linking. Considering the significantly higher vapor pressure of IPA and therefore faster evaporation compared to H_2_O, the pronounced shift of copolymer to the paper sheet surfaces can be explained. The observations furthermore indicate that the copolymer in solution does not appear to have interacted (electrostatically) with the molecular structure of the cellulose fibers in an attractive fashion (i.e., without having measured, we suspect a rather low enthalpy of adsorption), which would have affected the migration inside the paper sample. As shown by Wågberg and coworkers [29,40] electrostatic interactions between cellulose fibers and polymer molecules affect the spatial adsorption on the surface or throughout the fiber wall to a large extent. Attractive forces between the polymer and the cellulose fibers would have led to the adsorption of the macromolecules on/in the fibers, where they first came into sufficient contact. Reptation of the macromolecules after first adsorption has been observed for polyelectrolytes in cellulose fibers [29]; however, this is rather unlikely on the small timescales investigated here.

The effect of particle/chain size/molecular weight of the additive and the pore size distribution of the fibers must be taken into account due to possible size exclusion effects. Based on the determination of the hydrodynamic radii of the copolymer in H_2_O and IPA in combination with turbidity measurements, this effect could be ruled out, as shown in detail in our previous work [31]. In brief, an aqueous copolymer solution showed significant clouding even at the lowest concentration (5 mg mL^−1^) above 36 °C, while the dissolved copolymer in IPA showed no turbidity even at the highest concentration tested (45 mg mL^−1^) over a wide temperature range (5–50 °C). This led to the assumption that a size exclusion effect, which was of particular interest in the case of the IPA-dissolved copolymer, cannot explain the inhomogeneous distribution of the copolymer in the fiber network.

### 3.2. Drying-Induced Transport of the Copolymer in the Fiber Network

To investigate the transport of the polymer during drying, paper samples were impregnated with the copolymer out of the two solvents H_2_O and IPA, respectively. The concentrations of the solutions have been chosen in a way that yielded similar amounts of polymer in the paper samples after the modification. In detail, the concentration of the aqueous solution was 3.9 mg mL^−1^ (~1.1 wt%) and the concentration of the IPA-solution was 17 mg mL^−1^ (~1.9 wt%), respectively. The drying times after impregnation were varied before freeze-drying the samples in order to stop the evaporation. By that, the transport process was stopped before reaching an equilibrium state. By varying the drying times before freeze-drying and analyzing the distribution of the polymer in the thickness direction of the paper samples, the transport kinetics of the polymer throughout the fiber network was analyzed.

**Figure 5:**
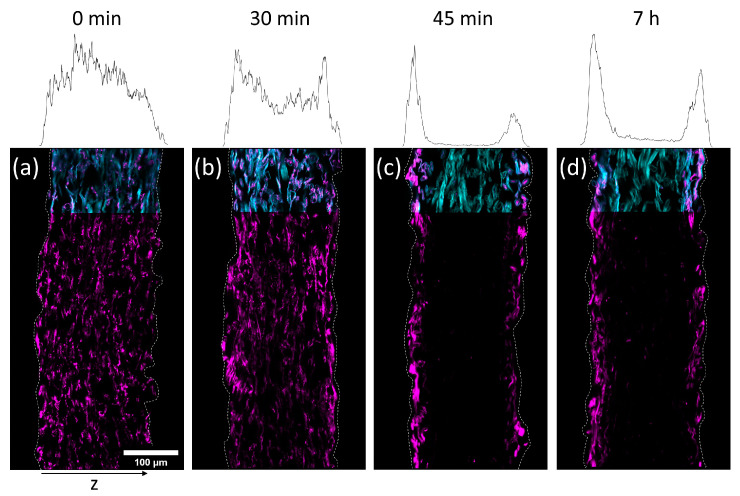
Cross-sections of paper samples modified with the photo-cross-linkable copolymer dissolved in H_2_O (3.9 mg mL^−1^), dried for different times (0 min (**a**); 30 min (**b**); 45 min (**c**); 7 h (**d**)) before freeze-drying to stop the evaporation (i.e., transport of the copolymer), acquired by fluorescence CLSM. The fibers are stained with FB (cyan color, outline continued) and the copolymer fluoresces due to the rhodamine B label (magenta). The left side of the images corresponds to the side that was facing upwards during the drying and UV-excitation of samples. The z-distribution of the copolymer fluorescence is shown above each cross-section.

**Figure 6:**
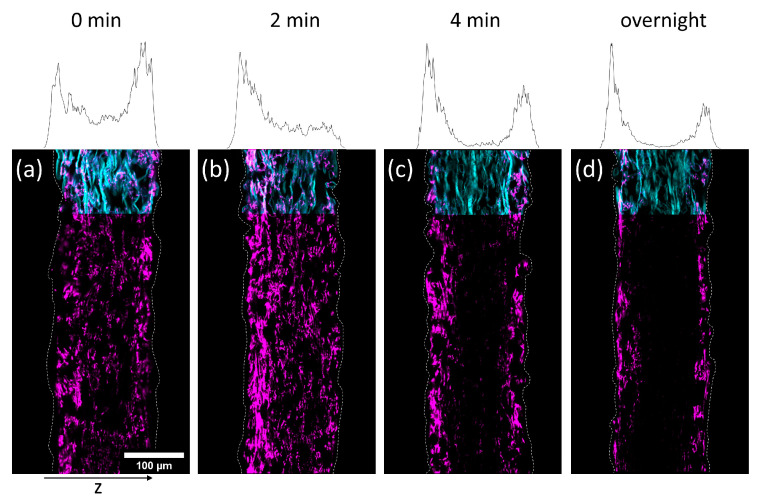
Cross-sections of paper samples modified with the photo-cross-linkable copolymer dissolved in IPA (17 mg mL^−1^), dried for different times (0 min (**a**); 2 min (**b**); 4 min (**c**); overnight (**d**)) before freeze-drying to stop the evaporation (i.e., transport of the copolymer), acquired by fluorescence CLSM. The fibers are stained with FB (cyan color, outline continued) and the copolymer fluoresces due to the rhodamine B label (magenta). The left side of the images corresponds to the side that was facing upwards during the drying and UV-excitation of samples. The z-distribution of the copolymer fluorescence is shown above each cross-section. For easier comparison, the corresponding drying times are also noted.

In Figure 5 and Figure 6 cross-sections of the impregnated paper samples analyzed by CLSM and the corresponding relative distribution of copolymer can be seen. In paper samples that were freeze-dried instantly after impregnation, the copolymer was distributed homogeneously throughout the paper thickness, regardless of which solvent had been used. With increasing drying times before freeze-drying the copolymer fluorescence was shifted towards the periphery of the samples. This effect was significantly faster for IPA compared to H_2_O-impregnation. After complete drying, the copolymer could only be observed at the periphery of the paper samples, i.e., top and bottom surfaces of the sheet. For IPA-impregnation, a stronger shift of copolymer fluorescence to the top side during drying was observed.

The results show that the copolymer was penetrating the whole paper sheet immediately after impregnation, regardless of the solvent used. During evaporation (i.e., drying) the polymer solution was transported from the bulk of the paper towards the top and bottom side, where the respective solvent was able to leave the paper by evaporation [41]. The transport process apparently has a significant impact on the copolymer deposition as well since the copolymer does not interact strongly with the cellulose fibers, i.e., the adsorption enthalpy is presumably not high enough to render the macromolecules in an adsorbed state during drying (i.e., transport) of the solvent. Hence, no retention was observed, confirming the previous observation. The latter behavior finally determines the distribution in the z-direction of the sheet. These findings underpin the results and interpretations of our previous experiments, where it was shown that the spatial distribution of polymer across the paper thickness is strongly dependent on the used solvent and the concentration of the solution used for impregnation [31].

Intuitively, IPA possessing significantly higher vapor pressure, thus evaporating faster, also shows a faster transport of copolymer to the periphery. For the complete range of CLSM analysis of all drying times, the reader is referred to Figures S4 and S5.

### 3.3. Different Drying Procedures to Achieve Designed Gradients in Paper Thickness

The results of the before discussed observations open the possibility to design paper samples with defined gradients of covalently fiber-attached polymer throughout the paper sheet thickness by adjusting the drying procedure. By using high temperatures and a solid surface on which the samples lie during drying, it should be possible to guide the transport of the polymer chains to one side. For this, paper samples of high grammage, namely 150 and 200 g m^−2^, were impregnated with the copolymer dissolved in H_2_O (34 mg mL^−1^) and IPA (48 mg mL^−1^) for 1 min. Afterward, the H_2_O-impregnated samples were put on a Teflon plate and dried in an oven at 120 °C for 0.5 h. The IPA-impregnated samples were dried lying flat on a Teflon surface without additional heating. After drying the samples were treated in analogy to the procedure described above.

**Figure 7:**
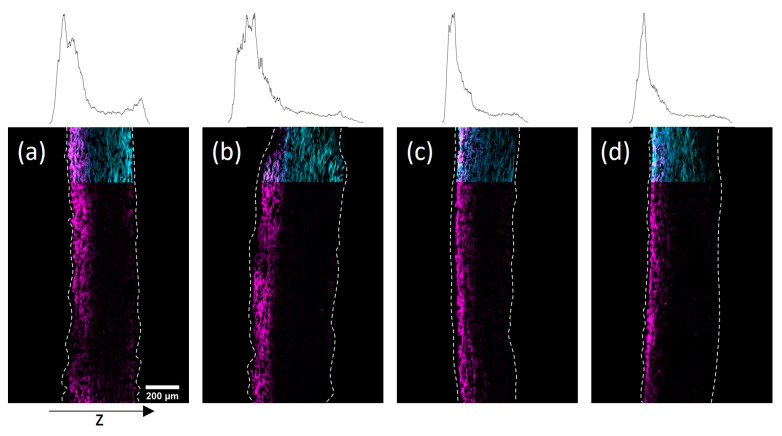
Cross-sections of paper samples with different grammages modified with the photo-cross-linkable copolymer dissolved in H_2_O (34 mg mL^−1^) dried in an oven at 120 °C on Teflon-plates (150 g m^−2^ (**a**); 200 g m^−2^ (**b**)), and dissolved in IPA (48 mg mL^−1^) dried on Teflon-plates at r.t. (150 g m^−2^ (**c**); 200 g m^−2^ (**d**)), acquired by fluorescence CLSM. The fibers are stained with FB (cyan color, outline continued) and the copolymer fluoresces due to the rhodamine B label (magenta). The left side of the images corresponds to the side that was facing upwards during drying and UV-excitation of samples. The z-distribution of the copolymer fluorescence is shown above each cross-section.

In Figure 7 cross-sections of the impregnated paper samples prepared by CLSM and the corresponding distribution of copolymer through the thickness of the sheets can be seen. Using the modified drying procedure, significant copolymer gradients to the top side could be achieved for both solvents. However, using IPA the gradients appeared significantly more pronounced, which was readily observable in the representation of the copolymer profile within the thickness direction. The observed polymer distribution in the thickness direction of the paper samples exemplifies the ability to design gradients by choosing a specific drying procedure.

**Figure 8:**
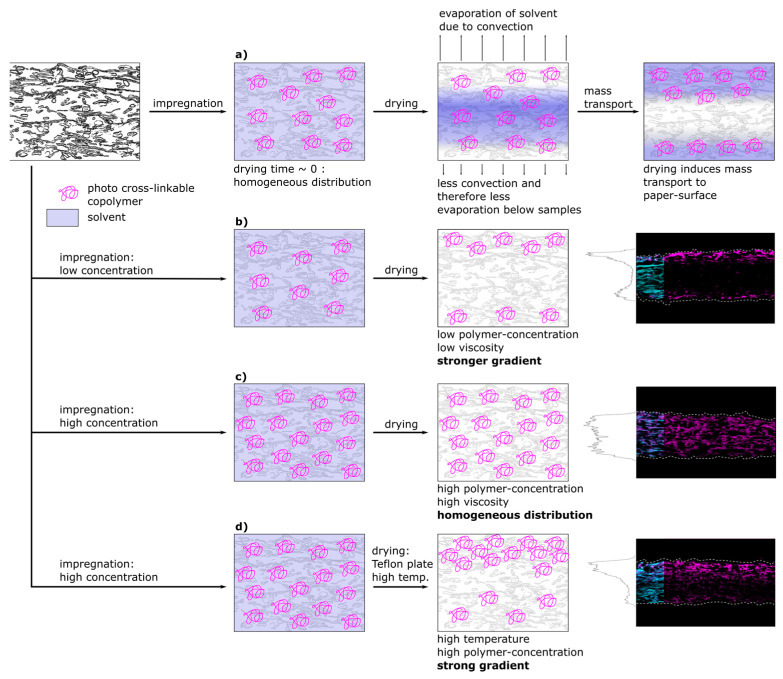
Model representation of a drying-induced transport in paper samples impregnated with copolymer solutions possessing different concentrations under various drying conditions, based on our conceptual understanding and the conducted experiments. For the cross-sections prepared by fluorescent CLSM the fibers are stained with FB (cyan color, outline continued) and the copolymer fluoresces due to the rhodamine B label (magenta).Upon impregnation the macromolecules are distributed homogeneously throughout the paper sample before being transported towards the surfaces during drying (**a**); conceptual representation at low (**b**) or high (**c**) polymer concentrations during impregnation and drying; combination of high concentration, increased temperature and limited drying (**d**).

Figure 8 shows a schematic representation of a proposed model that is able to explain the experimentally observed spatial distribution of polymer inside fiber networks. In the first part of Figure 8a the proposed spatial distribution of the copolymer after impregnation during the different steps of drying is depicted. Directly after impregnation, and prior to transport and drying, the copolymer macromolecules are distributed homogeneously throughout the whole paper thickness, which was also observed experimentally (see Figure 5 and Figure 6, respectively). After impregnation, the samples were dried at climate-controlled conditions (23 °C, 50% r.h.). Drying on a sieve with Teflon yarn allowed for convection and drying from both paper surfaces, however, the airflow under the sieve was limited. This led to an asymmetrical drying profile that is depicted and which has been observed experimentally by analyzing the copolymer distribution. During drying, the copolymer solution has been consecutively transported to the surfaces of the paper sample, where the solvent could evaporate. The copolymer subsequently precipitated, ultimately determining the copolymer distribution across the paper sheet thickness.

As our results suggest, there was no indication that the copolymer is interacting in an attractive fashion with the cellulose fibers. Instead, adsorption took place spontaneously upon the removal of the solvent (see Figure 5 and Figure 6). Without attractive interactions, one cannot assume similarities for the adsorption process analogous to the adsorption of charged macromolecules to cellulose fibers studied numerous times, showing adsorption isotherms until saturation is reached [18,26,27,28].

For both, low and high copolymer concentrations in the impregnation solution, the proposed model for the copolymer transport is depicted in Figure 8b,c, respectively. At low polymer concentration (b) before drying, the polymer is distributed homogeneously and during drying transported and accumulated at the surfaces of the paper sample. Increasing the copolymer concentration in the solution (Figure 8c) led to an increasingly homogeneous distribution of copolymer after drying (see Figure 3 and Figure 4). This can be explained by the viscosity of the polymer solution. The force acting against the transport along the drying-induced mass transport of solvent is the fluid friction, which is also known as viscous drag. With increasing polymer concentration, the viscosity of the solution and thus the fluid friction increases. This has been shown by [42] for aqueous solutions of poly(N,N-diethylacrylamide), which shows molecular similarities and thermoresponsivity (lower critical solution temperature) to poly(N,N-dimethylacrylamide) used in this study. At low concentrations the drying-induced mass transport is strong enough to overcome this friction. However, by increasing the concentration above a certain level, the friction exceeds the force of drying-induced transport. This hinders the polymer to be (fully) transported to the surfaces, and thus leads to earlier precipitation in the bulk of the network on the fiber surface. This led to an increasingly homogeneous distribution throughout the thickness as experimentally observed in Figure 3 and Figure 4. However, the distribution was not as homogeneous for the samples impregnated with IPA solutions compared to aqueous ones. This can be explained by the significantly different dissolution behavior of the polymer chains in the two solvents, as indicated by previous turbidity measurements [31].

A similar observation was made by [43] who observed the transport of aqueous solutions of methylhydroxyethylcellulose (MHEC) in a packed bed consisting of glass beads during drying. By conducting drying experiments and consecutive TGA analysis, they showed that the distribution shows a gradient increasing towards the top at increased concentrations of MHEC. This was explained by the increased viscosity of the MHEC solution inhibiting the transport to the top surface. At lower concentrations, they observed enrichment of the MHEC on the surface, which is analogous to the observations of the experimental results of this study.

In Figure 8d the model proposes the creation of sharp gradients across the thickness of paper samples at higher drying temperatures. High viscosity and therefore friction forces can be overcome by accelerating the mass transport of the solvent due to the increased temperature. Combining high drying temperature with drying on a Teflon plate allows the control of transport to only one side, because the evaporation to the bottom side is restricted. The resulting distribution of polymer for paper samples at higher paper grammages and polymer concentrations can be seen in Figure 7, even though at temperatures above the LCST the viscosity is significantly increased [42]. Interestingly, for IPA-impregnated samples, this could be achieved by restricting the drying direction at room temperature without increasing the temperature. This is another indicator for the proposed significant differences in the dissolution behavior of the copolymer in the two used solvents. Apparently, the copolymer dissolved in IPA precipitated at a significantly later stage during drying compared to aqueous solutions, thus allowing for further transportation towards the surfaces of paper samples even at higher concentrations.

Experiments to determine the adsorption characteristics of a labeled polyvinylamine polymer, that is positively charged at a neutral pH [44], showed a significantly different spatial distribution across the z-axis of impregnated paper sheets. In the cross-sectional images shown in Figure S6 the fluorescently labeled polyvinylamine can be observed homogeneously distributed across the whole thickness of the paper sheet, regardless of the drying time. Compared to the impregnation with the neutral photo cross-linkable copolymer, this observation points toward different adsorption and transport phenomena during drying. The cationically charged polyvinylamine was interacting in an attractive fashion with the cellulose fibers and due to unfavorable enthalpic change for desorption remained in an adsorbed state. Thus, the polyvinylamine was not transported to the surface of the impregnated paper samples during drying, in contrast to the photo-cross-linkable copolymer used. This has also been observed for paper sheets impregnated with CPAM, confirming the hypothesis that the charge and the attractive interactions prohibit transport during drying [20].

This work shows that by carefully choosing the procedure during impregnation and especially drying, directed transport of the polymer in the fiber network can be achieved. This opens up the possibility to design samples where the polymer is mainly located close to one or both sides of the paper surface or distributed homogeneously throughout the thickness.

### 3.4. Influence of Copolymer Gradients on the Tensile Properties

After having analyzed the spatial distribution of the copolymer applied to the paper samples by different strategies, we furthermore studied the influence of the distribution on the tensile properties of these samples. In analogy to the experiments conducted before, the solvents used were H_2_O and IPA and the concentrations of the solutions and therefore the amount of polymer in the paper samples was varied.

**Figure 9:**
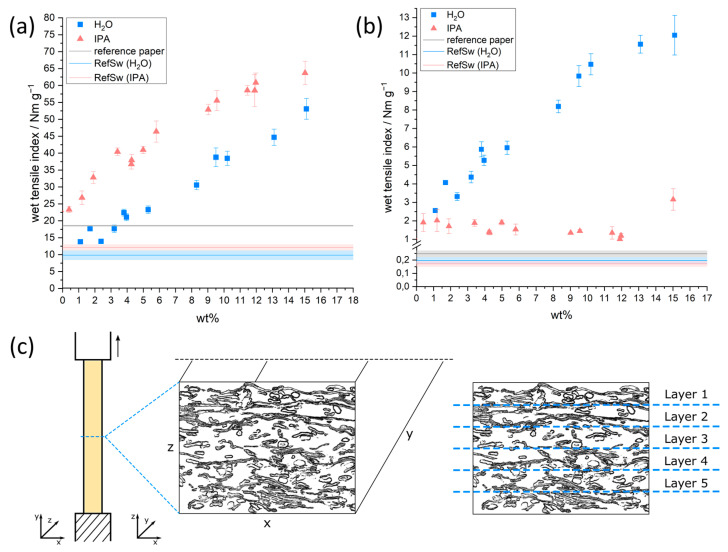
Dry (**a**) and wet (**b**) tensile index values of paper samples impregnated with copolymer solutions of varying concentrations and the two solvents H_2_O (blue squares) and IPA (peach triangles), respectively. As a point of reference, three tensile index values of pure reference eucalyptus paper (reference paper), and paper samples that were treated in analogy to the copolymer application, but without any polymer, in the solvents used for the initial swelling step with H_2_O (RefSw—H_2_O) and IPA (RefSw—IPA), respectively. (**c**) Schematic representation of a paper sheet during tensile testing, focusing on the “quasi-layers” of fiber–fiber bonds in the z-direction. The paper was sectioned into five layers in the thickness direction, in order to keep the image uncluttered.

Figure 9a,b shows the dry and wet tensile index as a function of the amount of copolymer in paper samples relative to the paper fiber weight. The model sheets were impregnated from H_2_O and IPA with varying concentrations, as described before. Figure S7 furthermore shows the calculated values for the relative wet strength.

If the photoreactive polymer was applied from H_2_O, both dry and wet tensile indices showed an almost linear increase with increasing concentration of covalently bound copolymer in the paper samples (blue squares). The dry tensile index increased from about 10 Nm g^−1^ (0 wt% polymer added relative to paper fiber mass) to about 53 Nm g^−1^ if ~15 wt% polymer is added and cross-linked in the sheet. At the same time, the wet tensile index increased from about 0.2 to about 12 Nm g^−1^. The latter corresponds to ~23% relative wet strength (see Figure S7). Similarly, if the polymer was applied from IPA the dry tensile index increased almost linearly with an increasing amount of added polymer to about 64 Nm g^−1^. Compared to the application from H_2_O, the resulting dry tensile index was higher when impregnation was carried out from IPA, while the wet tensile index was significantly lower, which can be observed over the entire range of concentrations used here. Similar observations were made for a single and distinct concentration of cross-linked polymer in the sheet [31]. This work does not focus on the distribution of polymer on a single fiber level and the influence on the resulting wet strength of the samples. Therefore, the interested reader is referred to our previous work.

Comparing the CLSM-images (see Figure 3, Figure 4, Figures S2 and S3) with the tensile measurements outlined before, it becomes apparent that strengthening of the samples took place, even if the copolymer was not distributed homogeneously throughout the whole paper thickness. This behavior can be explained by taking a closer look at the fiber network and the mechanism of failure (see Figure 9c). From a macroscopic view, a fiber network that is strained in one direction (*y*-axis) fails perpendicular (*x*-axis) to the loading direction. If the network would consist of one layer of fibers, every point of fiber-fiber interaction along the width of the network (*x*-axis) would have to fail before the whole sample would rupture. Since paper consists of multiple layers, depending on the basis weight/grammage, for complete rupture of the paper sample every one of these layers (*z*-axis) has to fail along the width of the network. Taking this into consideration, it becomes clear how even a thin continuous copolymer layer can lead to a significant strengthening of the paper samples, provided that there are no major “defects” or inhomogeneities present in this layer. With increasing polymer amount throughout the paper, the force before failure for the thicker copolymer layer will scale, as can be seen in the linear trend of the tensile values (see Figure 9). Similar results were reported for paper samples strengthened with the dry strength agent CPAM, applied by different methods, leading to inhomogeneous distribution throughout the thickness [20].

The analysis conducted here shows that the tensile properties of paper are mainly influenced by the amount of added photo-reactive copolymer rather than the macroscopic distribution across the thickness. While the wet tensile strength of paper samples impregnated with the copolymer from IPA was significantly lower compared to using aqueous solutions, it was nevertheless significantly increased compared to an unmodified paper sheet (0.17 to 1.32 Nm g^−1^). Therefore, both procedures led to paper samples with increased wet strength, which have been evaluated as humidity-responsive actuation devices.

### 3.5. Paper Actuator Demonstration

With the possibility to prepare wet strengthened paper samples with defined copolymer gradients across the thickness direction in situ, the ability of said samples to act as humidity-responsive actuators was evaluated. For this, high-grammage eucalyptus paper samples (150 g m^−2^) were prepared and subsequently modified with the copolymer from H_2_O (35 mg mL^−1^) and IPA (48 mg mL^−1^) with a modified drying procedure. The spatial distribution of the copolymer throughout these samples has already been shown in Figure 7a,c. The samples were fixed on one end inside a box, where the humidity was varied (see Figure 1) and a camera was used to observe the deflection of the samples.

**Figure 10:**
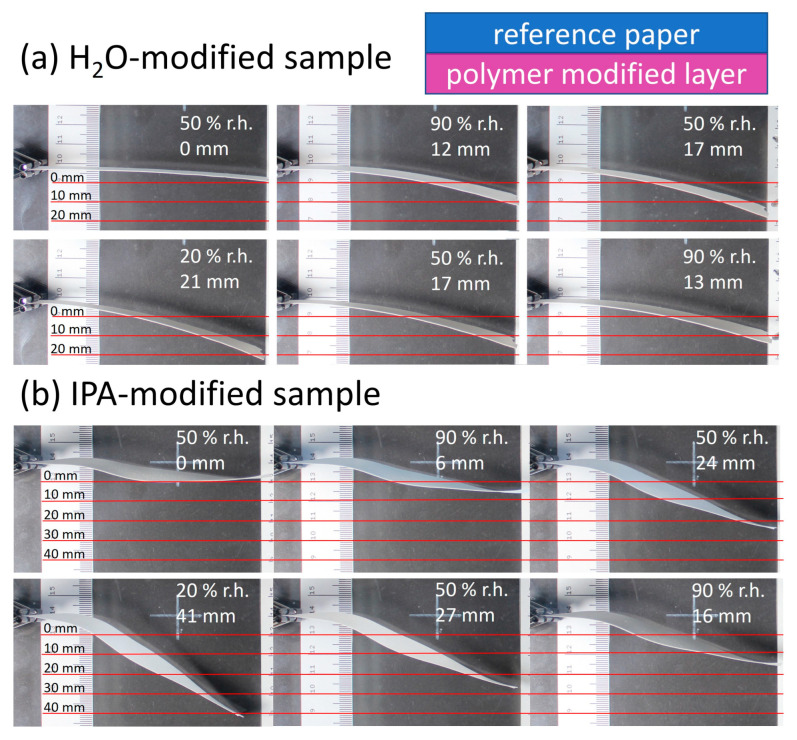
First humidity cycle showing the deflection of the humidity-responsive paper samples (eucalyptus 150 g m^−2^) with copolymer applied from (**a**) H_2_O and (**b**) IPA, polymer modified “layer” pointing down, in the closed chamber with semi-quantitative analysis of the change of deflection. After each humidity change, the samples were acclimated for 2 h to reach equilibrium.

Figure 10 shows the paper samples modified via the above-mentioned procedure, with their sides facing upwards during modification (polymer-modified “layer”) pointing toward the lower part of the images. Varying the humidity, significant deflection up- or downwards can be seen and analyzed semi-quantitatively using the scale incorporated in the images.

The relative humidity was varied from 90% to 20% to 90% for each sample. In the beginning, the samples were positioned horizontally in the chamber, in analogy to the orientation seen in Figure 1. The first deflection at 90% r.h. occurred towards the modified “layer” of the samples (lower part of images) and was 12 mm and 6 mm for the H_2_O- and IPA-impregnated samples, respectively. Reducing the humidity in the chamber led to more pronounced deflection reaching a maximum at 20% r.h. of 21 mm and 41 mm for the H_2_O- and IPA-impregnated samples, respectively. Comparing the change of deflection from high to low humidity, a higher responsivity was observed for the IPA-modified samples (35 mm), compared to the H_2_O-impregnated samples (9 mm). Paper samples without any modification showed no significant deflection, confirming the influence of the modification, as can be seen in Figure S8. Further experiments were conducted where the paper sample location in the PMMA box with controlled humidity was changed, to rule out any effects the airflow could have on the deflection. During these control experiments, the same observation regarding the deflection in the direction of the modified paper side was made.

**Figure 11:**
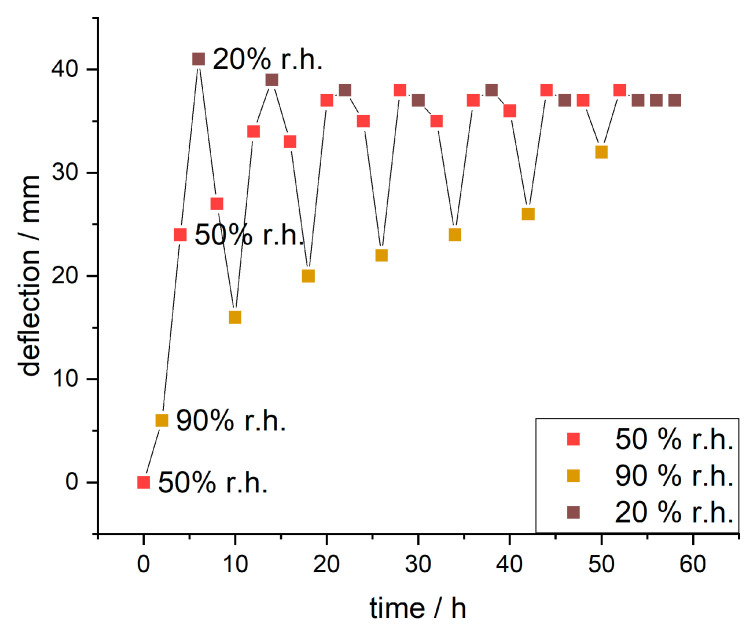
Deflection of a humidity-responsive paper sample (eucalyptus 150 g m^−2^ with copolymer from IPA) during changing r.h. over time. After each humidity change, the samples were acclimated for 2 h to reach equilibrium.

Since the IPA-modified samples showed significantly higher responsivity, these samples were further analyzed over multiple days with varying humidity (humidity curve in Figure S9). The evaluated deflections are plotted against the time in Figure 11.

During the first cycle, the paper sample showed deflection from 6 mm to 41 mm to 16 mm changing the humidity from 90% to 20% to 90% r.h., respectively. The observed maximum delta of deflection during this was 35 mm. With further cycles (full range of images in Figure S10) the deflection at 90% r.h. increased incrementally (from 6 to 32 mm), the maximum deflection at 50% r.h. stayed roughly the same and the deflection at 20% r.h. reduced slightly (from 41 to 37 mm). This resulted in decreasing delta values for deflection (from 35 to 5 mm).

The observed deflection can be explained by the observed gradient of the copolymer in the z-direction of the paper samples already shown in Figure 7. This has an effect on the hygroexpansion of the fiber network, which can basically be seen as a functional bilayer, with an unmodified and a modified “layer”. These layers can also be described as the “active” and “passive” resistance layer, as suggested by [45]. In an unmodified sheet of paper, an increase in humidity leads to the adsorption of moisture from the surrounding air until an equilibrium is reached. Decreasing the relative humidity leads to the consecutive desorption of water. It is reported that the moisture adsorption isotherm for paper has the shape of an S-curve and shows hysteresis effects [46]. In order to get a better understanding of the humidity–responsive behavior of the samples in this study, it is important to know how the morphology of cellulose fibers changes during water adsorption and desorption and what factors can affect these changes. Single fibers show a higher expansion in the transverse than in the longitudinal direction, leading to anisotropic swelling. This swelling results in significant dimensional changes also known as hygroexpansion in the thickness of paper sheets and to a lower degree in the in-plane direction. In-plane hygroexpansion is more important when discussing the deflection of the actuators prepared herein. Values for hygroexpansion in the plane of paper sheets without additives have been reported to be around 0.5% to 0.9% for free and restrained dried paper [47,48,49].

Impregnating the paper with the cross-linked PDMAA copolymer dissolved in IPA led to the polymer being distributed as relatively large agglomerates in the fiber network (see Figure S3). It behaved like a hydrogel, thus being able to adsorb and desorb moisture, which resulted in swelling and shrinking, as has been shown for thin surface-attached films of the copolymer [50].

**Figure 12:**
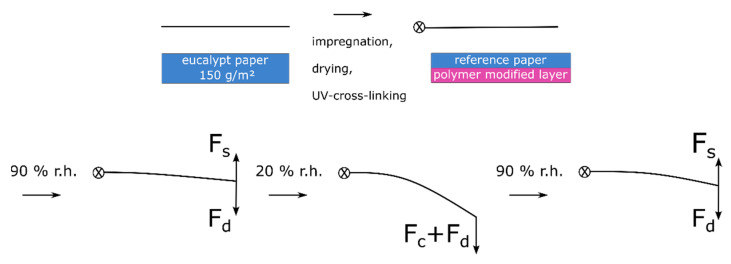
Schematic representation of the forces acting upon the single-layer paper actuator during copolymer impregnation, drying, pressing and upon exposing it to different relative humidity regimes. The forces represent the drying-induced residual stresses (F_d_) after impregnation and the forces resulting from dissimilar hygroexpansion of the unmodified resistance “layer” and the copolymer-modified active “layer” of paper due to the swelling (F_s_) and compaction (F_c_) of the active layer.

During the deflection experiments, the samples were first subjected to 90% r.h. before the humidity was lowered to 20% r.h. In the first adsorption cycle, the paper samples adsorbed moisture, though the adsorbed mass differed significantly for the unmodified (+14.14/+12.90 wt%) resistance “layer” and the polymer modified (+18.14/+16.74 wt%) active “layer”, as observed by dynamic vapor sorption measurements (see Figure S11 and Table S3). Assuming that the adsorption of moisture also leads to significant dimensional changes of the “layers”, a significant deflection away from the active copolymer layer (image top side) would be expected, though this was not the case here. This counterintuitive observation can be explained by considering all forces/stresses acting on the paper actuator, which are schematically shown in Figure 12.

From the above results, we postulate a hypothesis regarding the actuation scenarios and stress states of the paper-based material system: During the drying of the paper sample after impregnation in the copolymer solution, the solvent is transported to the surface, where it evaporates. This gradient of moisture during drying leads to forces acting in the fiber network ultimately resulting in residual stress (F_d_) in the paper sample; however, they are not observable macroscopically. This is in analogy to the description of moisture-induced deformation of paper sheets [51]. By photo-cross-linking the copolymer in the fiber network, this stress is irreversibly “programmed” into the fiber network. Before the samples are used as actuators they are pressed, to obtain relatively flat samples.

Upon an increase of humidity from 50% to 90% r.h. a slight deflection in the direction of the active “layer” (bottom image side) is observed. This can be explained by comparing the two acting forces of swelling hydrogel (F_s_) of the bottom layer and the force resulting from drying stress (F_d_). These forces are similar in their magnitude, leading to a slight deflection during adsorption. During the change from 90% to 50% and finally to 20% r.h. the layers desorb moisture. In the case of the hydrogel, this leads to a significantly larger compaction (F_c_) of the layer compared to the unmodified resistance layer, together with the residual drying stress resulting in deflection towards the active layer (bottom image side). Upon the next adsorption step, both layers adsorb moisture, which leads to swelling (F_s_), which is significantly larger for the active layer and results in the deflection of the actuator towards the resistance layer (top image side), i.e., towards its initial state. The force due to residual drying stress (F_d_) acts against the deflection, inhibiting a complete return to the initial state of the actuator.

Although we lack a complete physical understanding as to why the deflection is attenuated with each adsorption–desorption cycle, there are a couple of possible explanations for this. Similar behavior of paper-based actuators has been reported earlier [15] and can be explained by the changes in paper morphology, e.g., by hornification that decreases the moisture content adsorption and thus the dimensional change with every cycle [47]. The decrease in moisture uptake during the adsorption cycle results in decreased hygroexpansion (F_s_) acting against the dried-in stress (F_d_), therefore leading to less deflection. Furthermore, it is known that bending at high humidity leads to plastic deformation that could act against the swelling and deflection towards the initial state [51]. Another aspect is the hydrogel and the change of swelling properties after multiple swelling-drying cycles. It is conceivable that the hydrogel will behave differently, and this has an effect on the behavior of the fiber network.

These first demonstrations show that it is possible to create humidity-responsive actuators out of a single sheet of paper, without the need to incorporate multiple layers of different materials that were necessary for stimuli-responsive actuation in previous studies [13,52]. We see clear advantages in such one-sheet functional bi- or even multi-“layers”: (1) The risk of delamination, which is otherwise a threat for layered actuators during deformation cycles (cf. [53]), should be drastically lowered; (2) the resulting compliant systems can be produced in a thinner and, thus, more light-weight fashion; and (3) the construction complexity (and potentially) costs are lower. Interestingly, this paper-based material system highlighted here is a functional analogy to hygroscopic plant structures where tissue material gradients in one functional layer (rather than layers of different tissues) dictate differential hygro-actuated bending responses, like the stems of the false rose of Jericho (*Selaginella lepidophylla*) [54]. By smart compartmentalization of paper-based actuators, future approaches will presumably be capable of incorporating multiple actuator domains into one single-layered structure. Thereby, localized deformation sequences can be achieved and harnessed, e.g., for a biomimetic transfer of complex edge actuation scenarios as known from petals of the blooming lily (*Lilium* spec.) [55]. Moreover, the versatility of multi-layered motile plant structures should also be emphasized, since they can show a remarkable variety of reversible and repeatable motion sequences. A very good example of this is the pine cone, where four tissue types are involved in the hygroscopic motion and which have different functions by acting as a motor (sclereid cells), water barrier, and transducer (epidermises, brown tissue, and sclerenchyma strands) and as resistance layer with hydraulically switchable mechanical properties (sclerenchyma strands) [4]. This highlights the high potential for versatile biomimetic “motion programming”.

## 4. Conclusions

The adsorption behavior of the photo-cross-linkable fluorescent copolymer P(DMAA-co-MABP-co-RhBMA) dissolved in the two solvents H_2_O and IPA to cellulose fiber sheets/network/paper was studied. The polymer concentration as well as the drying method were found to have profound effects on the spatial distribution. By utilizing these effects and precisely choosing the application parameters it was possible to achieve fine-tuned polymer distribution gradients throughout the paper thickness. Drying on a Teflon plate restricts evaporation to one side of paper thus directing the transport of the polymer macromolecules in the fiber network. Tensile analysis of the modified paper sheets showed that a homogeneous distribution of cross-linked copolymer throughout the paper thickness isn’t necessary to enhance the dry and wet tensile index. Hence, our results show that it is more important to have a continuous layer without defects (in the plane of paper), rather than the layer spanning across the entire thickness of the sheet.

Such single layer paper sheets having gradients in fiber-attached polymers were proven to be interesting candidates for paper-based actuators driven by changes in humidity, mimicking plant structures such as the false rose of Jericho (*Selaginella lepidophylla*). Designing humidity-responsive actuators out of a single sheet of paper has several advantages over multi-layered structures, reducing the risk of delamination, and of the design and construction complexity. Finally, we observed a strong hysteresis if multiple deflection cycles were run. For an in-depth understanding of this behavior, possible reasons such as hornification, plastic deformation of the fiber network, as well as the polymer network will be addressed in follow-up studies.

## Data Availability

The data presented in this study are available in this published article and its supplementary material.

## Supplementary Materials

The following supporting information can be downloaded at: https://www.mdpi.com/article/10.3390/biomimetics8010043/s1, Figure S1: Example of a ^1^H NMR spectrum taken from the photo-cross-linkable copolymer P(DMAA-co-MABP-RhBMA). The aromatic protons of the benzophenone group and the fluorescent rhodamine B group are not distinguishable and labelled together, shown with a red overlay; Figure S2: Cross-sections of paper samples modified with the photo-cross-linkable copolymer dissolved in d. H_2_O at different concentrations, acquired by fluorescence CLSM. The fibers are stained with FB (cyan color) and the copolymer fluoresces due to the rhodamine B label (magenta). The top side of the images corresponds to the side that was facing upwards during drying and UV-excitation of samples. In addition, magnified insets are included to enable the analysis of polymer distribution across the fiber width and the lumen. Scale bars are 50 µm (large images) and 20 µm (small inserts), respectively. For easier comparison the corresponding concentrations of polymer solutions and the gravimetrically determined amount of copolymer is also noted; Figure S3: Cross-sections of paper samples modified with the photo-cross-linkable copolymer dissolved in IPA at different concentrations, acquired by fluorescence CLSM. The fibers are stained with FB (cyan color) and the copolymer fluoresces due to the rhodamine B label (magenta). The top side of the images corresponds to the side that was facing upwards during drying and UV-excitation of samples. In addition, magnified insets are included to enable the analysis of polymer distribution across the fiber width and the lumen. Scale bars are 50 µm (large images) and 20 µm (small inserts), respectively. For easier comparison the corresponding concentrations of polymer solutions and the gravimetrically determined amount of copolymer is also noted; Figure S4: Cross-sections of paper samples modified with the photo-cross-linkable copolymer dissolved in d. H_2_O (3.9 mg mL^−1^), dried for different times before freeze-drying to stop the evaporation (i.e., transport of the copolymer), acquired by fluorescence CLSM. The fibers are stained with FB (cyan color, outline continued) and the copolymer fluoresces due to the rhodamine B label (magenta). The left side of the images corresponds to the side that was facing upwards during drying and UV-excitation of samples. The z-distribution of the copolymer fluorescence is shown above each cross-section. For easier comparison the corresponding drying times are also noted; Figure S5: Cross-sections of paper samples modified with the photo-cross-linkable copolymer dissolved in IPA (17 mg mL^−1^), dried for different times before freeze-drying to stop the evaporation (i.e., transport of the copolymer), acquired by fluorescence CLSM. The fibers are stained with FB (cyan color. outline continued) and the copolymer fluoresces due to the rhodamine B label (magenta). The left side of the images corresponds to the side that was facing upwards during drying and UV-excitation of samples. The z-distribution of the copolymer fluorescence is shown above each cross-section. For easier comparison the corresponding drying times are also noted; Figure S6: Cross-sections of paper samples modified with the fluorescently labelled poly vinyl amine dissolved in d. H_2_O (5 mg mL^−1^), dried on a Teflon sieve at ambient conditions for different times before freeze-drying to stop the evaporation (i.e., transport of the polymer), acquired by fluorescence CLSM. The fibers are stained with FB (cyan color) and the polymer fluoresces due to the rhodamine B label (magenta). The left side of the images corresponds to the side that was facing upwards during drying of samples; Figure S7: Relative wet strength values of paper samples impregnated with copolymer solutions of varying concentrations and the two solvents H_2_O (blue squares) and IPA (peach triangles), respectively. As a point of reference, three tensile index values of pure reference eucalyptus paper (reference paper), and paper samples that were treated in analogy to the copolymer application, but without any polymer, in the solvents used for the initial swelling step with H_2_O (RefSw—H_2_O) and IPA (RefSw—IPA), respectively; Figure S8: Paper sample without any modification inside the humidity controlled PMMA-box at 90 and 20% r.h., respectively, where no significant deflection is observable; Figure S9: Relative humidity curve during the actuation experiment over multiple adsorption and desorption cycles. The measured humidity in the PMMA box at every desorption cycle reached ~20% r.h.; Figure S10: Full range of humidity cycles showing the deflection of the humidity-responsive paper sample in the closed chamber with semi-quantitative analysis of the deflection change. After each humidity change, the samples were acclimated for 2 h to reach equilibrium; Figure S11: Dynamic vapor sorption measurements of eucalypt sulphate paper with 100 g/m² unmodified and modified with the copolymer applied out of IPA, respectively; Table S1: Copolymer concentration of aqueous solutions used for impregnating paper samples and the gravimetrically determined amount of copolymer, in relation to the dry paper weight; Table S2: Copolymer concentration of IPA-solutions used for impregnating paper samples and the gravimetrically determined amount of copolymer, in relation to the dry paper weight; Table S3: Dynamic vapor sorption measurement results of eucalypt sulphate paper with 100 g/m^2^ unmodified and modified with the copolymer applied out of IPA, respectively.
