# Supplementary material for: Chemical Gradients in Polymer-Modified Paper Sheets—Towards Single-Layer Biomimetic Soft Robots
Source: Biomimetics (Basel). 2023 Jan 18;8(1):43. doi: 10.3390/biomimetics8010043 (PMC9944451; doi:10.3390/biomimetics8010043)
Supplement: Supplementary file 1 [file biomimetics-08-00043-s001.zip › biomimetics-2146660-supplementary.pdf]

Supporting Information

Chemical Gradients in Polymer-Modified Paper Sheets—Towards Single-Layer Biomimetic Soft Robots

Jan-Lukas Schäfer [1], Tobias Meckel [1], Simon Poppinga [2] and Markus Biesalski [1,*]

[1] Department of Chemistry, Macromolecular Chemistry & Paper Chemistry, Technical University of Darmstadt, Alarich-Weiss-Straße 8, 64287 Darmstadt, Germany
[2] Department of Biology, Botanical Garden, Technical University of Darmstadt, Schnittspahnstraße 10, 64287 Darmstadt, Germany
* Correspondence: markus.biesalski@tu-darmstadt.de; Tel.: +49-6151-1623721

^{1}H-NMR analysis of copolymers used

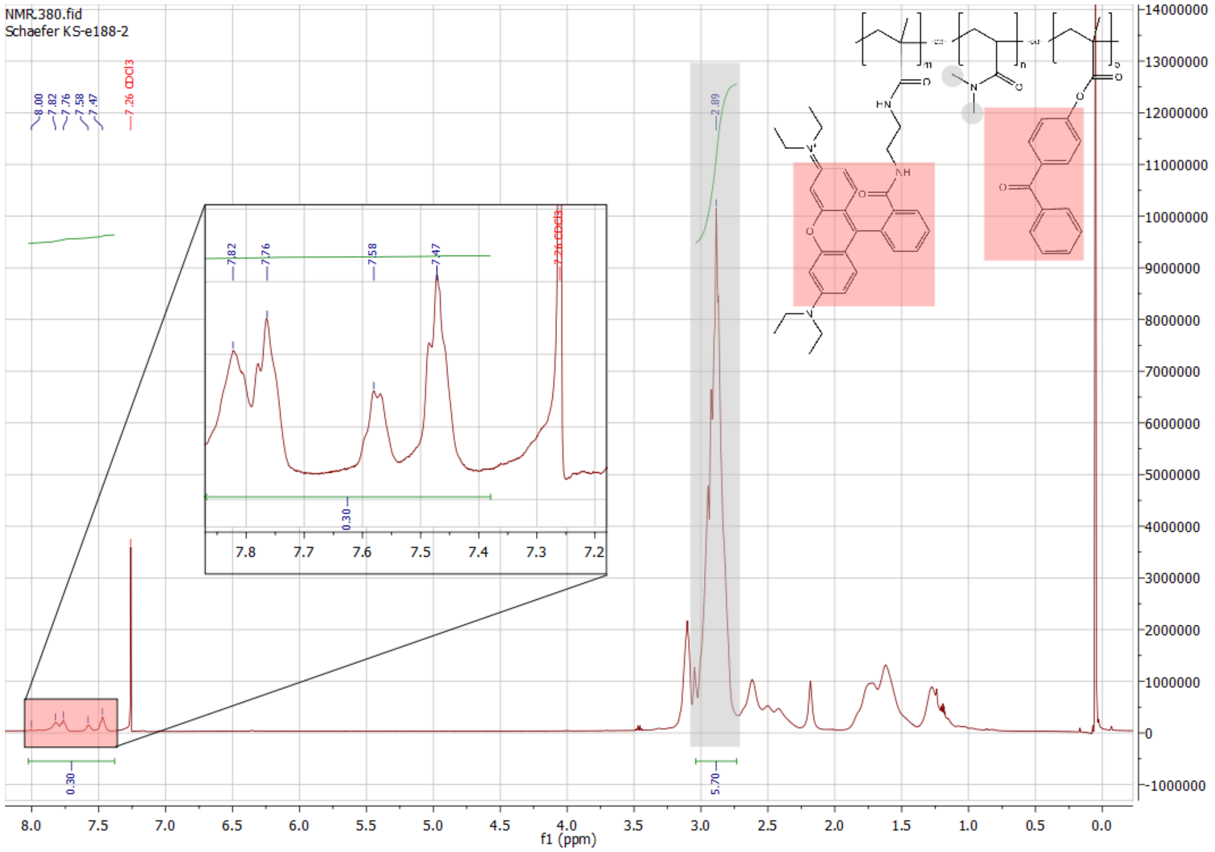

Figure S1 Example of a ^{1}H NMR spectrum taken from the photo-cross-linkable copolymer P(DMAA-co-MABP-RhBMA). The aromatic protons of the benzophenone group and the fluorescent rhodamine B group are not distinguishable and labelled together, shown with a red overlay.

Gravimetric results after impregnating paper samples

In the following tables the used concentrations of copolymer with the accompanying gravimetrically determined amount of copolymer, in relation to the dry weight of the paper samples, are listed for aqueous and IPA-solutions, respectively.

Table S1 Copolymer concentration of aqueous solutions used for impregnating paper samples and the gravimetrically determined amount of copolymer, in relation to the dry paper weight.

Concentration mg/mL	Copo amount / wt%
0	0
0	0
4.6	1.1
9.3	1.7
10.2	2.4
13.2	3.2
16.6	4.0
18.9	3.8
20.2	5.3
25.7	9.5
29.9	8.3
35.3	10.2
43.5	13.1
50	15.1

Table S2 Copolymer concentration of IPA-solutions used for impregnating paper samples and the gravimetrically determined amount of copolymer, in relation to the dry paper weight.

Concentration mg/mL	Copo amount / wt%
0.0	0.0
0.0	0.0
5.0	0.4
10.5	1.2
18.4	1.9
19.3	4.3
19.3	4.3
20.8	3.4
28.2	5.0
30.7	5.8
38.2	9.0
39.1	9.6
44.0	12.0
49.7	11.4
50.0	11.9
55.6	15.0

<u>**Analysis of microscopic copolymer distribution in fibers and fiber network**</u>

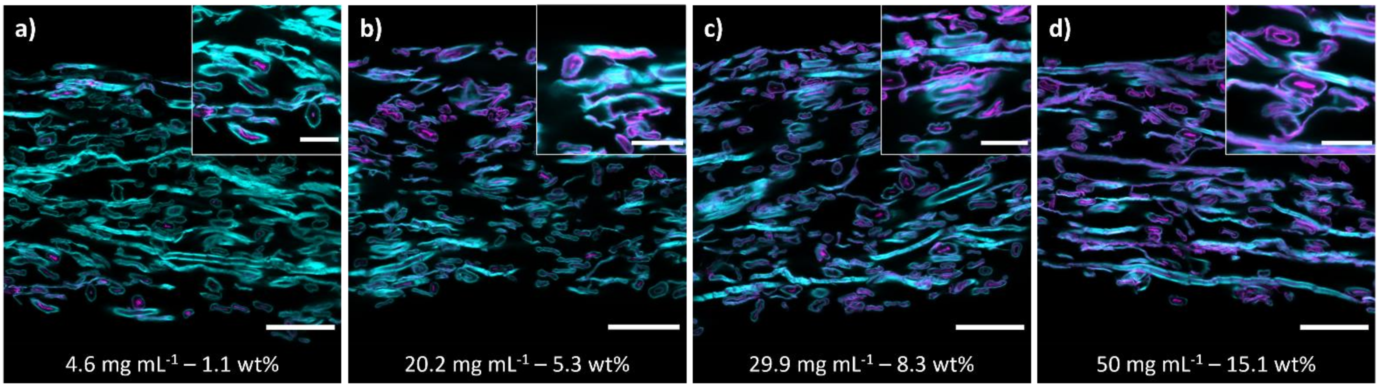

Figure S2 Cross-sections of paper samples modified with the photo-cross-linkable copolymer dissolved in d. H_2O at different concentrations, acquired by fluorescence CLSM. The fibers are stained with FB (cyan color) and the copolymer fluoresces due to the rhodamine B label (magenta). The top side of the images corresponds to the side that was facing upwards during drying and UV-excitation of samples. In addition, magnified insets are included to enable the analysis of polymer distribution across the fiber width and the lumen. Scale bars are 50 µm (large images) and 20 µm (small inserts), respectively. For easier comparison the corresponding concentrations of polymer solutions and the gravimetrically determined amount of copolymer is also noted.

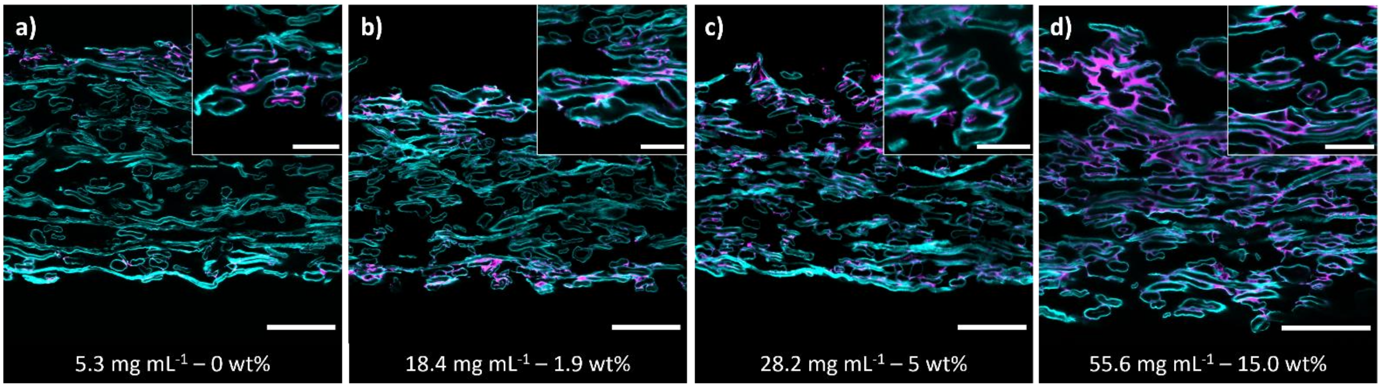

Figure S3 Cross-sections of paper samples modified with the photo-cross-linkable copolymer dissolved in IPA at different concentrations, acquired by fluorescence CLSM. The fibers are stained with FB (cyan color) and the copolymer fluoresces due to the rhodamine B label (magenta). The top side of the images corresponds to the side that was facing upwards during drying and UV-excitation of samples. In addition, magnified insets are included to enable the analysis of polymer distribution across the fiber width and the lumen. Scale bars are 50 µm (large images) and 20 µm (small inserts), respectively. For easier comparison the corresponding concentrations of polymer solutions and the gravimetrically determined amount of copolymer is also noted.

Analysis of copolymer transport during drying for the solvents d. H_2O and IPA

In the following figures S4 + S5 the polymer distribution inside paper samples (80 g/m²) after impregnation with copolymer solutions out of d. H_2O or IPA before freeze-drying for various times is shown. In addition to the small set of samples shown in the manuscript, here the full investigated range of drying times before freeze-drying can be seen.

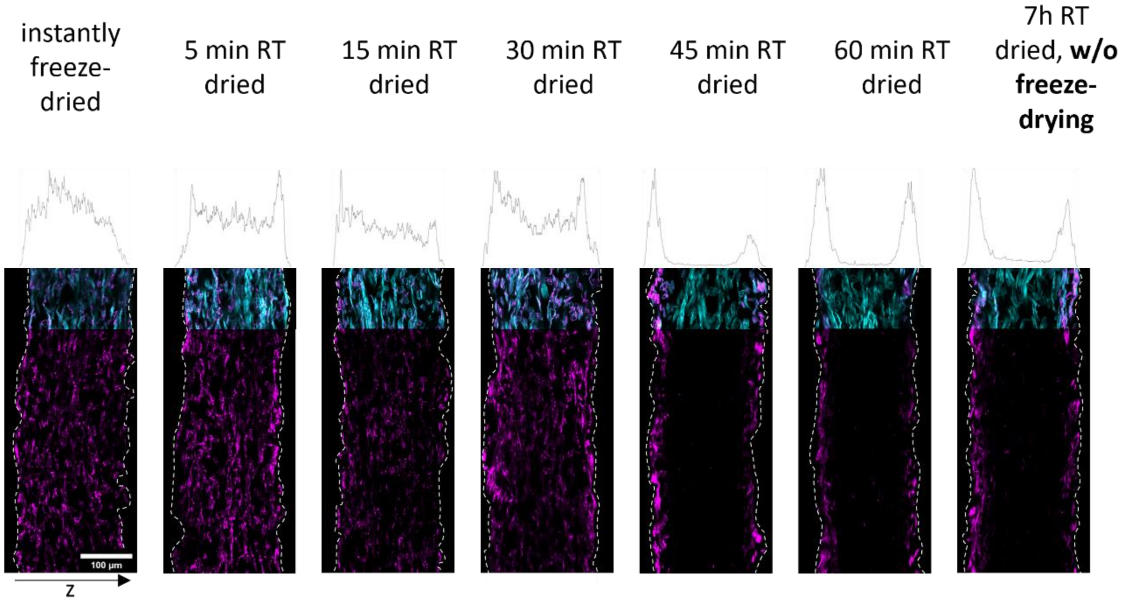

Figure S4 Cross-sections of paper samples modified with the photo-cross-linkable copolymer dissolved in d. H_2O (3.9 mg mL^{-1}), dried for different times before freeze-drying to stop the evaporation (i.e., transport of the copolymer), acquired by fluorescence CLSM. The fibers are stained with FB (cyan color, outline continued) and the copolymer fluoresces due to the rhodamine B label (magenta). The left side of the images corresponds to the side that was facing upwards during drying and UV-excitation of samples. The z-distribution of the copolymer fluorescence is shown above each cross-section. For easier comparison the corresponding drying times are also noted.

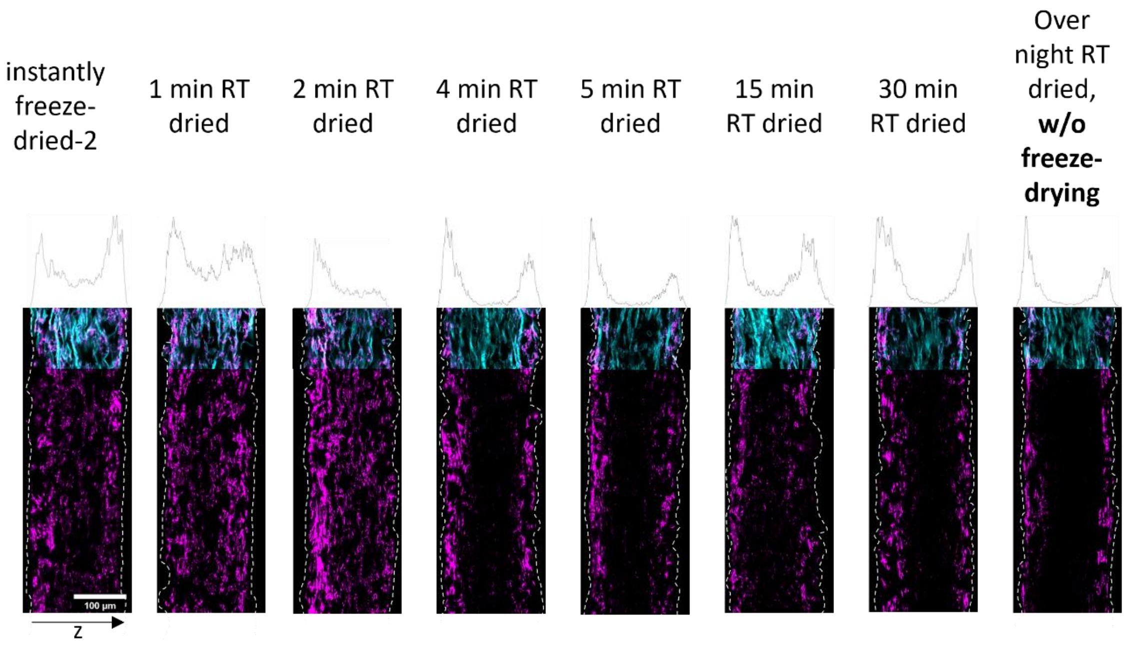

Figure S5 Cross-sections of paper samples modified with the photo-cross-linkable copolymer dissolved in IPA (17 mg mL^{-1}), dried for different times before freeze-drying to stop the evaporation (i.e., transport of the copolymer), acquired by fluorescence CLSM. The fibers are stained with FB (cyan color. outline continued) and the copolymer fluoresces due to the rhodamine B label (magenta). The left side of the images corresponds to the side that was facing upwards during drying and UV-excitation of samples. The z-distribution of the copolymer fluorescence is shown above each cross-section. For easier comparison the corresponding drying times are also noted.

Paper samples 80 g/m² with poly vinyl amine solution in d. H_2O 5 mg/mL

Paper samples were treated in analogy to impregnation with the cross-linkable copolymer with an aqueous solution of rhodamine b labelled poly vinyl amine (5 mg/mL) after which they were frozen in l. N_2 and subsequently freeze-dried to stop the polymer transport in the fiber network. Afterwards cross-sections of the paper samples were prepared for CLSM and the results are shown here (fig. S6).

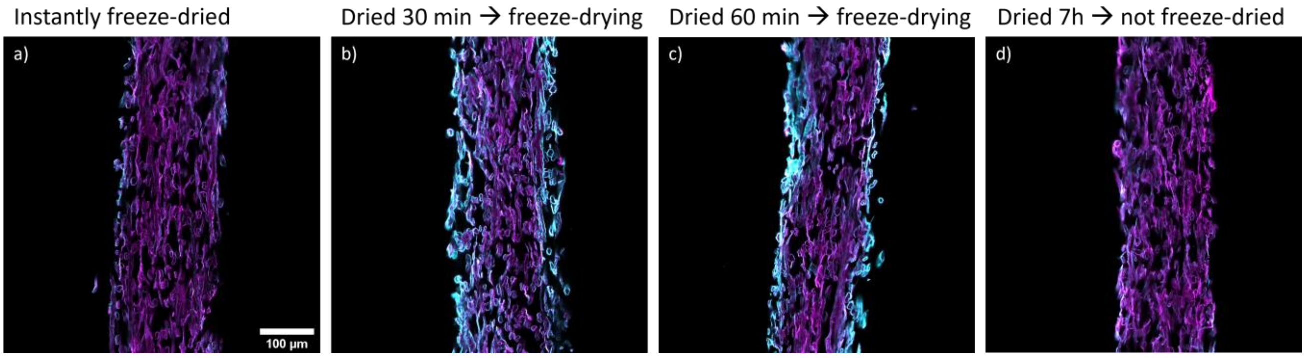

Figure S6 Cross-sections of paper samples modified with the fluorescently labelled poly vinyl amine dissolved in d. H_2O (5 mg mL^{-1}), dried on a Teflon sieve at ambient conditions for different times before freeze-drying to stop the evaporation (i.e., transport of the polymer), acquired by fluorescence CLSM. The fibers are stained with FB (cyan color) and the polymer fluoresces due to the rhodamine B label (magenta). The left side of the images corresponds to the side that was facing upwards during drying of samples.

Tensile testing – reference measurements – influence of swelling-drying-cycles

For comparison purposes it is important to treat the samples in a similar manner to the ones modified with the photo-cross-linkable copolymer, in order to take the effects of swelling-drying-cycles on the tensile strength into account. It has been found multiple times that swelling and drying of cellulose fiber sheets leads to a decrease in tensile strength which can be explained by fiber hornification (ref. [~~1~~47] in main manuscript). The same is also observed for the reference paper samples investigated in this study (see fig. 9).

Figure S7 shows the relative wet strength for the paper samples impregnated with the two solvents H_2O and IPA with different amounts of polymer, calculated from the measured dry and wet tensile index values.

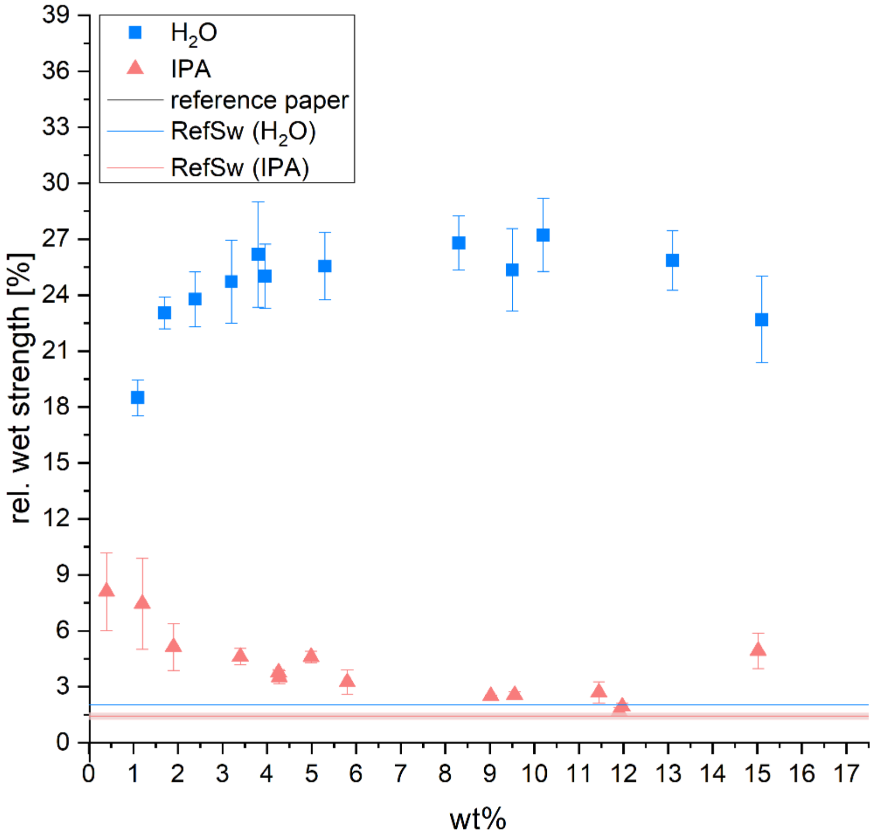

Figure S7 Relative wet strength values of paper samples impregnated with copolymer solutions of varying concentrations and the two solvents H_2O (blue squares) and IPA (peach triangles), respectively. As a point of reference, three tensile index values of pure reference eucalyptus paper (reference paper), and paper samples that were treated in analogy to the copolymer application, but without any polymer, in the solvents used for the initial swelling step with H_2O (RefSw – H_2O) and IPA (RefSw – IPA), respectively.

Humidity-responsive actuation behavior of single layer copolymer-modified paper sheets

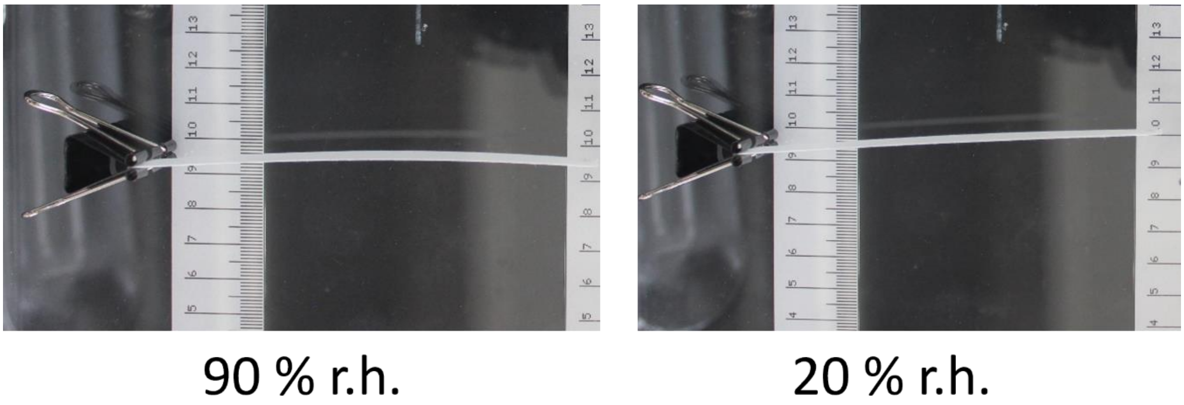

Figure S8 Paper sample without any modification inside the humidity controlled PMMA-box at 90 and 20 % r.h., respectively, where no significant deflection is observable.

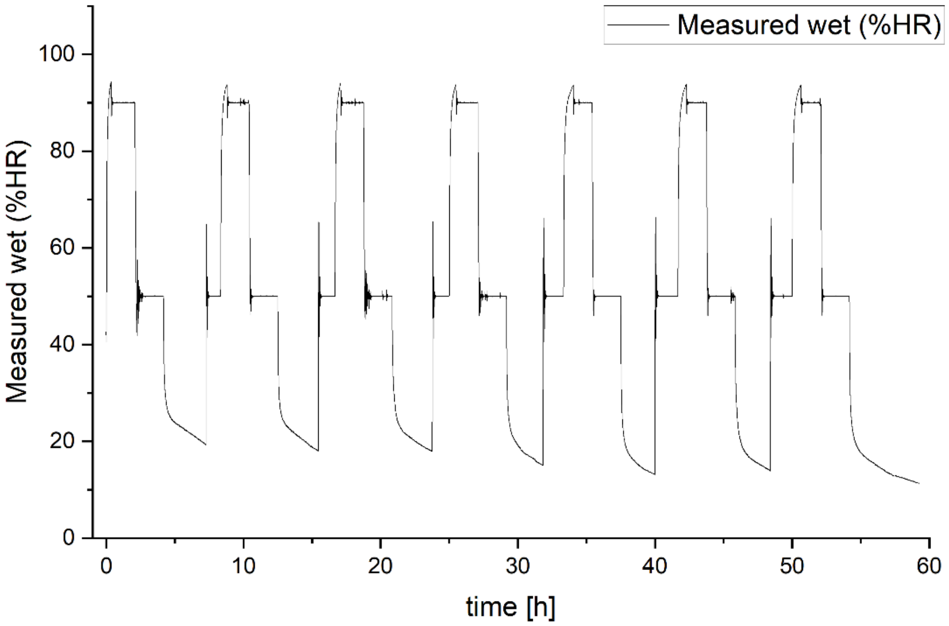

Figure S9 Relative humidity curve during the actuation experiment over multiple adsorption and desorption cycles. The measured humidity in the PMMA box at every desorption cycle reached ~20 % r.h.

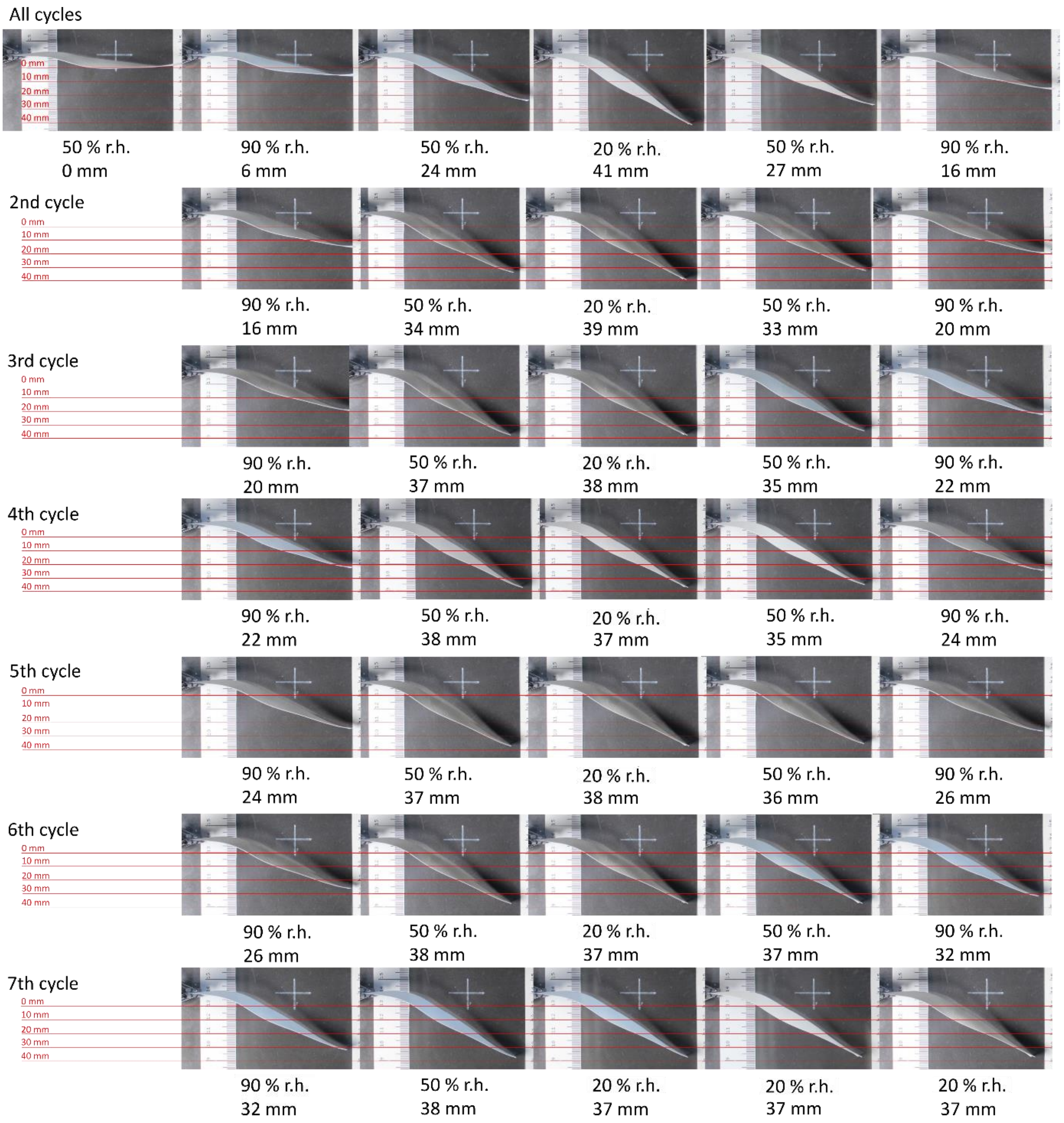

Figure S10 Full range of humidity cycles showing the deflection of the humidity-responsive paper sample in the closed chamber with semi-quantitative analysis of the deflection change. After each humidity change, the samples were acclimated for 2 h to reach equilibrium.

Dynamic vapor sorption measurements of unmodified and copolymer-modified paper samples

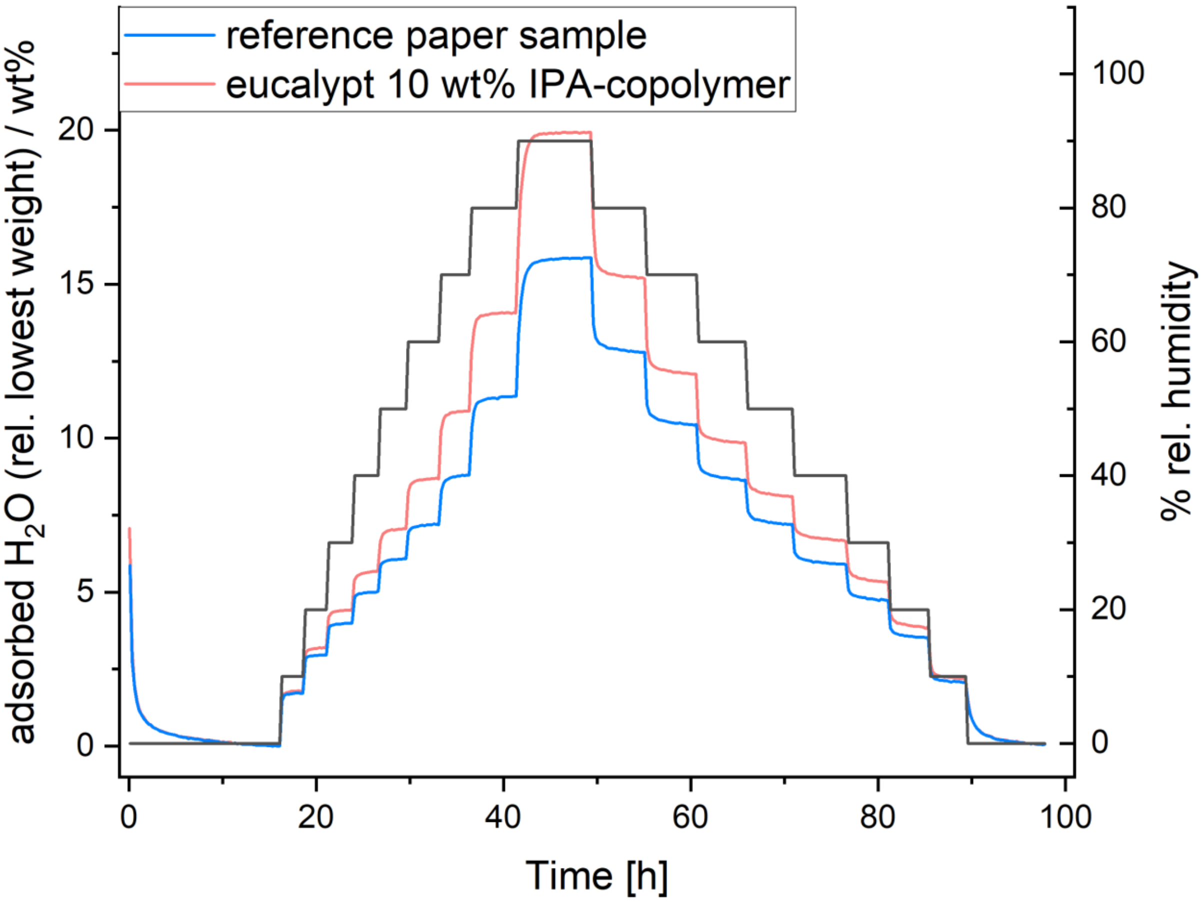

Figure S11 Dynamic vapor sorption measurements of eucalypt sulphate paper with 100 g/m² unmodified and modified with the copolymer applied out of IPA, respectively.

Table S3 Dynamic vapor sorption measurement results of eucalypt sulphate paper with 100 g/m² unmodified and modified with the copolymer applied out of IPA, respectively.

	Reference eucalypt 100 g/m²	IPA-Copolymer 10 wt%
**RH read at equili-brium**	**moisture wt%**	**moisture wt%**
**%RH**	**wt%**	**wt%**
1.5	0	0.00183
10.01	1.71744	1.7992
**19.97**	**2.95622**	**3.19614**
29.97	3.99778	4.40292
39.99	5.0188	5.67735
50	6.07885	7.05601
59.99	7.19231	8.67053
69.95	8.80703	10.88113
79.94	11.36471	14.06813
**89.92**	**15.85759**	**19.93564**
80.02	12.78838	15.18897
69.98	10.44025	12.0806
60.01	8.64885	9.85354
50.03	7.19642	8.10737
39.99	5.90628	6.65557
30.02	4.73324	5.31715
**20.02**	**3.52322**	**3.87449**
10.09	2.08106	2.18866
1.56	0.05341	0.06034

1. Sakaemura, T.; Yamauchi, T. Strength Properties of Paper Containing Polyacrylamide-Based Dry Strength Resin—Effect of Its Z-Directional Distribution. *Appita Technol. Innov. Manuf. Environ.* **2011**, *64*, 331.
